# A patient-designed tissue-engineered model of the infiltrative glioblastoma microenvironment

**DOI:** 10.1038/s41698-022-00290-8

**Published:** 2022-07-29

**Authors:** R. C. Cornelison, J. X. Yuan, K. M. Tate, A. Petrosky, G. F. Beeghly, M. Bloomfield, S. C. Schwager, A. L. Berr, C. A. Stine, D. Cimini, F. F. Bafakih, J. W. Mandell, B. W. Purow, B. J. Horton, J. M. Munson

**Affiliations:** 1 Department of Biomedical Engineering, University of Massachusetts Amherst, Amherst, MA 01003 USA; 2 Department of Biomedical Engineering & Mechanics, Virginia Tech, Blacksburg, VA 24061 USA; 3 Department of Biomedical Engineering, University of Virginia, Charlottesville, VA 22904 USA; 4 Fralin Biomedical Research Institute, Virginia Tech, Roanoke, VA 24016 USA; 5 Department of Biological Sciences and Fralin Life Sciences Institute, Virginia Tech, Blacksburg, VA 24061 USA; 6 University of Virginia School of Medicine, Charlottesville, VA 22903 USA; 7 Department of Pathology, University of Virginia, Charlottesville, VA 22903 USA; 8 Department of Neurology, University of Virginia, Charlottesville, VA 22903 USA; 9 Department of Public Health Sciences, University of Virginia, Charlottesville, VA 22903 USA

**Keywords:** Cancer models, CNS cancer, Cancer stem cells, Translational research, Predictive markers

## Abstract

Glioblastoma is an aggressive brain cancer characterized by diffuse infiltration. Infiltrated glioma cells persist in the brain post-resection where they interact with glial cells and experience interstitial fluid flow. We use patient-derived glioma stem cells and human glial cells (i.e., astrocytes and microglia) to create a four-component 3D model of this environment informed by resected patient tumors. We examine metrics for invasion, proliferation, and putative stemness in the context of glial cells, fluid forces, and chemotherapies. While the responses are heterogeneous across seven patient-derived lines, interstitial flow significantly increases glioma cell proliferation and stemness while glial cells affect invasion and stemness, potentially related to CCL2 expression and differential activation. In a screen of six drugs, we find in vitro expression of putative stemness marker CD71, but not viability at drug IC_50_, to predict murine xenograft survival. We posit this patient-informed, infiltrative tumor model as a novel advance toward precision medicine in glioblastoma treatment.

## Introduction

Glioblastoma (GBM) is the most common and malignant form of primary brain cancer. While clinical treatments have advanced slowly over the last 25 years, introduction of the Stupp protocol (surgical resection, radiation, and oral temozolomide chemotherapy) established the current median survival of GBM patients at 15 months^1^. One difficulty in treating GBM is the diffuse invasion into surrounding tissue, where tumor cells acquire therapy resistance mediated by microenvironmental factors^2–4^. Identifying drugs to overcome this resistance and kill invaded cells has proven challenging. Drug screens of tumor cells alone on tissue culture plastic can be particularly limited since these are a poor representation of the tumor or invaded brain. Spheroid cultures have more complexity and recreate tumor geometry, but monocellular spheroids overlook elements like interactions with stromal cells, space for diffuse tumor spread, and biophysical forces found within the tissue.

The tissue surrounding a tumor, known as the tumor microenvironment (TME), contains cellular and extracellular factors that contribute to cancer progression^5,6^. In GBM and other cancers, the cellular TME can enrich cancer stem cell populations and increase tumor cell survival, proliferation, invasion, and drug resistance^7^. The brain TME is particularly unique because it contains cells specific to the central nervous system, such as astrocytes and microglia. In addition, we and others have shown that the biophysical force known as interstitial fluid flow increases during tumorigenesis and stimulates tumor cell invasion^8–11^. Recreating the multifaceted elements of the TME in experimental model systems can be difficult, and so orthotopic xenografts are the primary way to study these elements in combination. However, animal models are rather expensive, offer little control over the experimental variables, and ultimately may not capture human patient heterogeneity as well as expected^12^.

Tissue-engineered models of cancer offer substantial control, tunability, and cost-effectiveness as higher throughput screening tools compared to animal models. Furthermore, three-dimensional (3D) culture systems can approximate the in vivo tissue environment through incorporation of relevant stromal cells, extracellular matrices, and mechanical cues. Such complex models have been reported for breast, ovarian, and pancreatic cancer^13–15^, but most GBM models focus on modeling one element of the TME at a time^16,17^. One recent study reported on the co-culture of astrocytes, microglia, and tumor cells in a 2D format^18^; however, the geometry of the microenvironment is critical for recreating cellular states found in human GBM^19^. Furthermore, most models are based on arbitrary ratios of tumor cells to other cells, but our recent work established a need to use patient-relevant cellular ratios because the composition of the invasive brain tissue predicts patient survival^20^.

Here, we report the rational design of a 3D in vitro model of the human GBM TME incorporating patient-derived GBM stem cells (GSCs), human astrocytes and microglia, and interstitial fluid flow. The cellular ratios are defined from invasive margins of patient resection samples, and the interstitial flow rate is based on previous measurements in small animals^21^. Our model uses a hyaluronan-based matrix, the primary extracellular matrix component of the brain, and a tissue culture insert format for screening drug therapies at a physiologically relevant rate of tissue perfusion. We can simultaneously examine glioma cell invasion, death, as well as phenotypic markers for proliferation, putative stemness, and glial cell activation. This model and analysis therefore provides a holistic assessment of how the TME influences GBM malignancy and vice versa. Specifically, we use the expression of Ki67 as a marker of proliferating cells and CD71, or transferrin receptor 1, as a previously established marker of stem-like properties in glioma cells^21^. By assessing these glioma cell outcomes in our TME model, we examine (1) individual and synergistic effects of the cellular and biophysical GBM microenvironment on patient-derived glioma stem cell outcomes, (2) bidirectional intercellular communication between glioma and glial cells, and (3) correlations between in vitro drug response and prediction of in vivo murine xenograft survival.

## Results

### Invasive regions primarily contain neural astrocytes and microglia

**Fig. 1:**
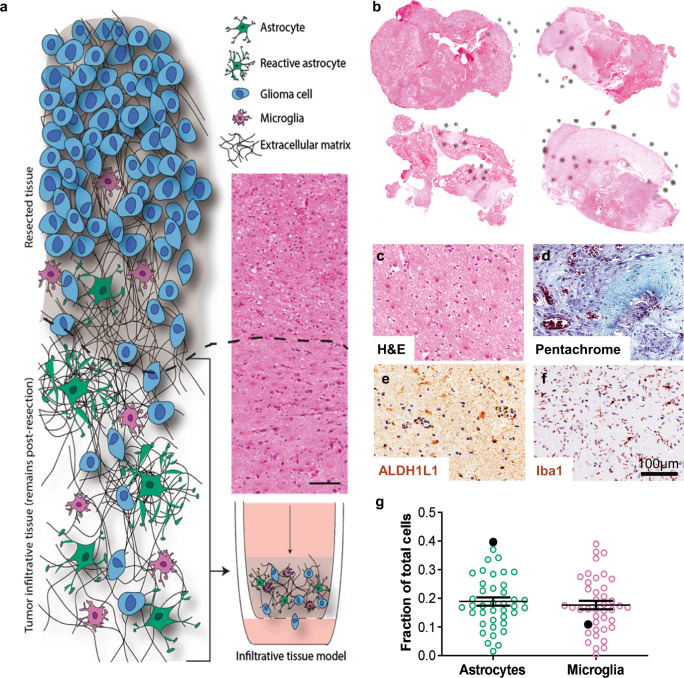
Histological quantification of the invasive human glioblastoma microenvironment for in vitro model development. **a** Illustration of the invasive tumor border and the patient-driven approach to develop a relevant model. **b** Representative bright-field scans of patient resection samples stained with hematoxylin and eosin (H&E), with dashed circles showing tumor-adjacent regions identified by a neuropathologist. **c**–**f** Representative bright-field images of chromogenic stains on serial patient samples for H&E (**c**), movat pentachrome (matrix and mucin staining, **d**), ALDH1L1 (astrocytes, **e**), and Iba1 (microglia, **f**). **g** Cell number quantification from our patient cohort samples (*N* = 40) for astrocytes and microglia, represented as a fraction of total nuclei count. Solid black circles show data from a select patient.

We analyzed the cellular composition of GBM patient resection samples toward developing a model of the brain TME (Fig. 1a). Using hematoxylin and eosin (H&E) stained slides, a neuropathologist determined that 40 of the 63 acquired samples contained sufficiently large regions of tumor-adjacent “reactive areas” (Fig. 1b) where invasive tumor cells reside. While no current cell marker is available to label and identify all GBM cells, it is possible to identify several other cell types of the microenvironment using immunohistochemical staining. Movat pentachrome staining also provides information about the extracellular matrix as well as cell-surface mucins. Representative images of H&E, Movat pentachrome, and chromogenic staining are shown in Fig. 1c–f. In serial sections, tumor-adjacent reactive areas contain approximately an equal fraction of astrocytes (ALDH1L1+) and microglia (Iba1+) at 18–19% each (Fig. 1g), though high variability exists between patient samples. In addition, approximately 75% of the reactive area contains neurons (~1%), oligodendrocytes (~16%), and general cell-surface mucins (~58%) (Supplementary Table 1). Given the need to assess in serial sections as opposed to the same sections, the total cellular composition equals 125% instead of 100%. We are primarily interested in evaluating metrics for tumor cells, and the exact glial cell fraction did not correlate with patient survival (Supplementary Fig. 1); therefore, we decided to keep 75% as the non-glial compartment (i.e., tumor cells) and set the ratio of astrocytes to microglia as 1:1 within the remaining fraction. Our final ratio used to build a model of the GBM TME is therefore 75:12.5:12.5 or 6:1:1 for glioma:astrocyte:microglia.

### Glioma stem cells remain viable and can be reisolated from 3D tri-culture

**Fig. 2:**
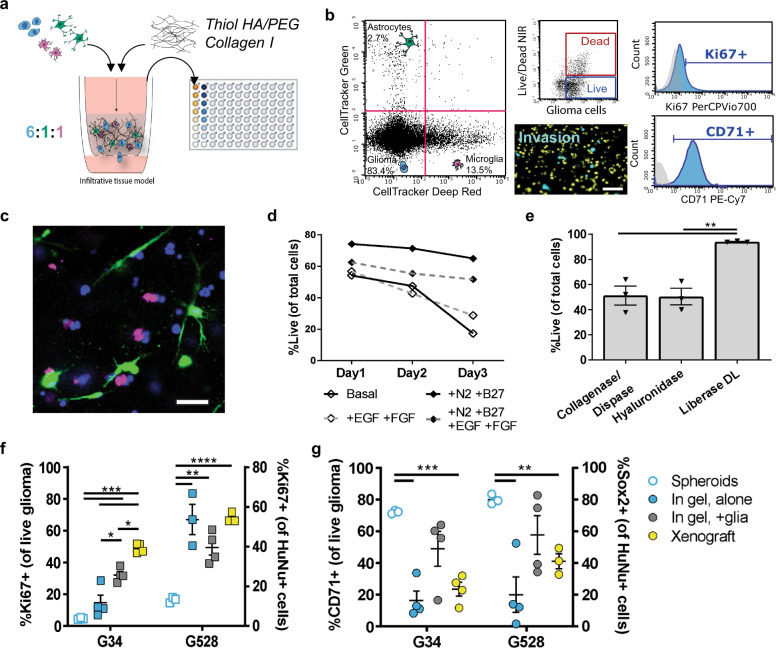
Tunable model of the human invasive TME enables multiplexed analysis of glioma markers and comparison to in vivo marker expression. **a** Diagram of brain TME model setup using patient-derived GSCs, human astrocytes, and human microglia in a hyaluronan-based matrix and 96-well tissue culture insert format. **b** Representative flow cytometry plots showing the ability to distinguish astrocyte, microglia, and glioma cell populations and determine glioma-specific proliferation (Ki67^+^), stemness (CD71^+^), and cell death. Isotype controls are shown in gray. Also shown is a representative fluorescence image of the porous membrane for invasion quantification. Scale bar is 50 µm. **c** Representative fluorescence image within the gel with glioma cells (blue), astrocytes (green), and microglia (magenta). Scale bar is 50 µm. **d** Quantification of cell viability under different media formulations for up to three days in hydrogel culture. **e** Quantification of cell viability following different enzymatic gel degradation protocols. **f**, **g** Comparison of %Ki67^+^ cells (**f**) and %CD71^+^ cells (**g**) for three in vitro cancer models and in vivo xenograft implants. In vitro data obtained by flow cytometry; in vivo data obtained from tissue sections, with the tumor border visually demarcated based on nuclear staining. Legends in **f**, **g** are the same. Comparisons conducted by unpaired *t*-tests, *p < 0.05 and **p < 0.01 for *n* = 3–4.

We used the cellular quantifications to develop a 3D tri-culture model of the human GBM TME comprising patient-derived GSCs, primary human astrocytes, and immortalized human microglia. The cells are embedded into a hyaluronan-based hydrogel and pipetted into a 96-well tissue culture insert (Fig. 2a). The porous membrane on the bottom of the insert enables the application of interstitial fluid flow via a pressure head of fluid on top (Supplementary Fig. 2). The hyaluronan-based hydrogel was previously optimized for mechanical properties and interstitial flow rates measured in the rodent brain^22^. Furthermore, the presence of interstitial flow enables the application of therapeutics in a manner mimicking physiological drug delivery. We designed the model to be compatible with simultaneous analysis of metrics by flow cytometry, such as proliferation (Ki67^+^), expression of putative stemness markers (CD71^+^), and cell death, as well as quantification of invasion by fluorescence microscopy (Fig. 2b and Supplementary Fig. 3a). Alternatively, the gels can be subjected to immunocytochemistry for additional analyses (Fig. 2c).

We first optimized the media formulation to achieve high glioma cell viability in the tri-culture gel format using a Cell Counting Kit-8 (CCK-8). We tested supplementing with epidermal growth factor (EGF) and fibroblast growth factor-2 (FGF-2b), two growth factors used for glioma stem cell maintenance, as well as N2 and B27 without vitamin A, two supplements optimized for neural cell culture. Astrocyte basal medium plus N2 and B27 yielded the highest cell viability for GSCs alone and in tri-culture for up to 3 days (Fig. 2d). We similarly optimized post-experiment harvesting of cells from the gels for subsequent analysis by flow cytometry. We tested three enzyme formulations for hydrogel degradation, and Liberase DL maintained glioma cell viability the best (Fig. 2e). We therefore chose this enzyme for all further experiments. We also evaluated live cell labeling using CellTracker or Vybrant dyes for downstream cell type identification and analysis, and the cells remain viable for a range of tested dyes (Supplementary Fig. 3b–d).

### Glioma cell expression of Ki67 and CD71 in tri-culture model mimics xenografts

Using the optimized model parameters, we compared phenotypic markers of tumor cells in the tri-culture model (without flow) to that in GSC monocultures and tumor-invaded regions of orthotopic xenografts. The percentages of GSCs in vitro with proliferative (Ki67^+^) and stem-like (CD71^+^) markers were evaluated by flow cytometry (using gating strategy shown in Supplementary Fig. 4), while in vivo proliferation (Ki67^+^) and stemness (Sox2^+^) were evaluated on tissue sections within regions of invasion of xenografted tumors (Supplementary Fig. 5). Sox2 was used as the marker for in vivo staining instead of CD71 because it is a nuclear marker and therefore easier to identify and quantify in expressing cells. Furthermore, CD71 and Sox2 are known to co-localize on GSCs in vivo^21^. We also compared the expression to two monocultures: non-adherent GSCs spheroids and GSCs encapsulated into the hydrogel model alone (no glial cells). After overnight culture in the three in vitro models tested—namely spheroids, in gel alone, and in gel+glia (astrocytes and microglia, no flow)—cells in spheroid culture exhibited a significant under-representation of proliferation and significant over-representation of stem-like properties compared to xenografted, invaded cells (*t* = 24.88, *p* < 0.0001 for G34 spheroid vs xenograft Ki67) (Fig. 2f, g). The hydrogel-based models tended to capture expression of these particular markers in vivo, with only G34 having significantly different expression in vitro versus in vivo. The importance of glial cells in this response varies by marker and GSC line.

### Glioma cell invasion is patient specific and depends on the TME context

**Fig. 3:**
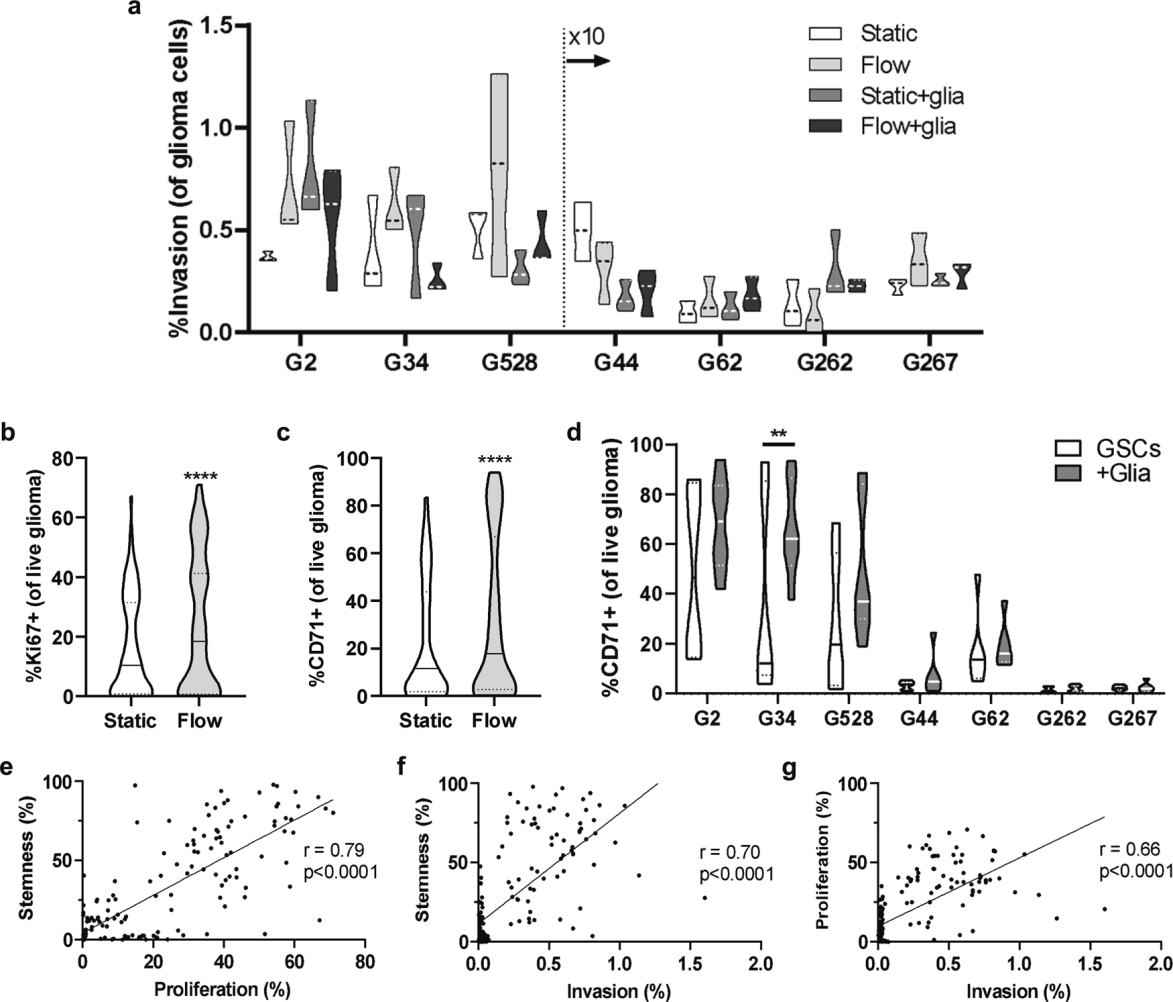
Interstitial flow induces the largest effect on glioma cell proliferation and stemness, and these metrics correlate with invasion. **a** Invasion data for each cell line in the presence and absence of interstitial fluid flow and/or glia (astrocytes and microglia). Each sample represents *n* = 3 technical replicates for *n* = 3 biological replicates. Data for G44, G62, G262, and G267 are multiplied by 10 to enable plotting on the same axis. **b** Ki67 expression data for all GSCs collected for all conditions (+astrocytes, microglia, or both) in static (white) vs in flow (light gray). **c** CD71 expression data for all GSCs collected for all conditions (+astrocytes, +microglia, or +both) in static (white) vs in flow (light gray). **d** CD71 expression data for each GSC line in static and flow when cultured alone (white) and in the presence of the TME (medium gray). Statistics were performed by paired *t*-tests with *p < 0.05, **p < 0.01, and ****p < 0.0001. **e** Correlation plot and linear regression of stemness (CD71^+^) vs. proliferation (Ki67^+^). **f** Correlation plot and linear regression of stemness (CD71^+^) vs. invasion. **g** Correlation plot and linear regression of proliferation (Ki67^+^) vs. invasion. The respective Pearson coefficients are shown in each plot for **e**–**g**.

To understand how glial cells and also fluid flow in the TME influence glioma cell outcomes, we expanded our analysis to include more GSC lines. We tested a total of seven GSC lines derived from different patients. Supplementary Table 2 shows a summary of known relevant properties for each patient cell line. We used our tunable, hydrogel-based model of the glioma TME to test how the GSC lines (G2, G34, G44, G62, G262, G267, or G528) are affected by transport condition (static or +flow) and glia (no glia, +astrocytes, +microglia, or +both) with respect to invasion, proliferation, and putative stemness. The collective results are plotted in Fig. 3a (and shown with more resolution as a heat map in Supplementary Fig. 6). It is also possible to tune the glial cell ratio to recreate specific patient data (Supplementary Fig. 7), but here we chose to focus on the case of the average TME cell ratio.

To assess the effects of glia and interstitial flow, we use a pressure head of medium on top of the gel to initiate flow through the matrix at approximately 0.54 µm/s^22^. We find interstitial flow enhances the invasion of five out of seven cell lines when the GCSs are cultured alone, but the response varies once glia are added to the model (Fig. 3a and Supplementary Table 3). For example, flow increases invasion of G34 cells when cultured alone, but the addition of astrocytes and microglia decreases G34 invasion under flow. With other cells—like G62—the effects of flow and glia are summative. Interestingly, the lines G2, G34, and G528 consistently show 10-fold higher baseline invasion than the other lines, underscoring the heterogeneity across these patient samples. To account for this variability, we employed a quantile regression model of the covariates accounting for skewness of the outcomes (Table 1). This approach enables simultaneous examination of the contribution of each model variable (GSC, transport, glia) to each outcome (invasion, proliferation, and stem-like expression) for determining which parameter significantly predicts the outcomes.

**Table 1:** Quantile regression predictive modeling of covariates in the in vitro TME model.

	Invasion			Proliferation		Stemness	
Covariate	Degrees of freedom	_*Χ*_2	*p* value	_*Χ*_2	*p* value	_*Χ*_2	*p* value
GSC	6	**83.97**	**<0.0001**	**36.22**	**<0.0001**	8.95	0.1766
Transport	1	0	0.9629	0.7	0.4027	0	0.9997
Glia	3	0	1.0000	1.18	0.757	1.23	0.745
GSC × Transport	6	10.57	0.1025	**29.81**	**<0.0001**	**64.68**	**<0.0001**
GSC × Glia	18	**38.59**	**0.0032**	27.15	0.0762	**86.62**	**<0.0001**
Transport × Glia	3	0	1.0000	1.54	0.6731	0.55	0.9067
GSC × Transport × Glia	18	**29.16**	**0.0464**	21.99	0.2324	**40.26**	**0.0019**

The individual or interacting covariates that reach statistical significance are shown in bold. “GSC” refers to glioma stem cells; “Transport” signifies conditions with interstitial fluid flow; and “Glia” signifies conditions with astrocytes and/or microglia. All analyses were performed using JMP software.

The GSC line is the only standalone covariate to significantly contribute to the percent of glioma cell invasion (*Χ*^2^ = 82.97.59, *p* < 0.0001). Therefore, inter-patient differences are the greatest contributor to invasion, and the presence of glial cells or fluid flow alone do not have generalizable effects across all lines. Nonetheless, the interaction between GSC line and glia (*Χ*^2^ = 38.59, *p* < 0.01), as well as glia and transport (*Χ*^2^ = 29.16, *p* < 0.05), significantly influence GSC invasion. In other words, percent invasion is specific to each patient line, but the addition of glial cells or a combination of glia and fluid flow also meaningfully influences GSC invasion either positively or negatively.

### Proliferation response is sensitive to cell line or flow

Proliferation of tumor cells is likely to be a major contributor to the aggressiveness of patient tumors. We find the percentage of proliferating glioma cells is highly dependent on the patient GSC line (*Χ*^2^ = 36.22, *p* < 0.0001) as well as how the cell line interacts with fluid flow (*Χ*^2^ = 29.81, *p* < 0.0001) (Table 1). Proliferation increases under flow compared to static conditions for two lines, namely G2 (*t* = 3.47, *p* < 0.001) and G34 (*t* = 3.5, *p* < 0.001), but proliferation significantly decreases under flow for G44 (*t* = −2.45, *p* < 0.001) and G267 (*t* = −2.28, *p* < 0.05) (Supplementary Fig. 6). The presence of glial cells does not significantly contribute to glioma cell proliferation across all lines, but it does within single lines. For example, G62 shows increased proliferation with the addition of either astrocytes or microglia under flow, but proliferation significantly decreases when both glia are present under flow (*t* = −1.99, *p* < 0.05). Collectively, fluid flow significantly increases glioma cell proliferation across all lines and microenvironmental conditions (Fig. 3b).

### Stem-like populations respond to each element of the TME model

The presence of cancer stem cells can be an important indicator of tumor growth and recurrence^23^. We assessed putative stemness based on expression of CD71, a marker previously identified to label glioma stem cells^21^. Figure 3c shows the presence of fluid flow significantly increases the percent of stem-like populations compared to the baseline static control across all lines (*p* < 0.0001). The presence of glial cells shows individualized effects by cell line (Fig. 3d), but the cellular TME significantly increases the percent of GSC stem-like populations across all lines together (*p* < 0.0001). Ultimately, we find expression of the stem-like marker CD71 is significantly modeled by the interaction of GSC line with each of the components: transport (*Χ*^2^ = 64.68, *p* < 0.0001), glial cells (*Χ*^2^ = 86.62, *p* < 0.0001), and all three covariates together (*Χ*^2^ = 40.26, *p* < 0.0019) (Table 1). There are also statistically significant interactions between transport and glia conditions. The changes in CD71 expression vary in effect size and direction across the cell lines, suggesting expression of this putative stemness marker might be a sensitive metric for evaluating the response of GSCs to variables such as cellular components and biophysical factors.

Next we sought to determine if invasion, proliferation, and stem-like expression in response to the TME elements correlate across the patient GSC lines, because infiltrative and drug-resistant cells in GBM may be slower-cycling and more stem-like^24^. To understand how these outcomes interrelate, we conducted correlation analyses for the outcomes across all conditions. Putative stemness and proliferation displayed a strong positive correlation with each other (Fig. 3e, Pearson *r* = 0.79, *p* < 0.0001). CD71 expression also displayed strong positive correlation with invasion (Fig. 3f, *r* = 0.70, *p* < 0.0001) as did proliferation, though the effect was moderate (Fig. 3g, *r* = 0.66, *p* < 0.0001). Importantly, these effects are all positively correlated, indicating these three “malignancy” metrics may increase similarly regardless of the experimental parameters.

### Pro-tumorigenic effects of the glioma TME are driven by CCL2

**Fig. 4:**
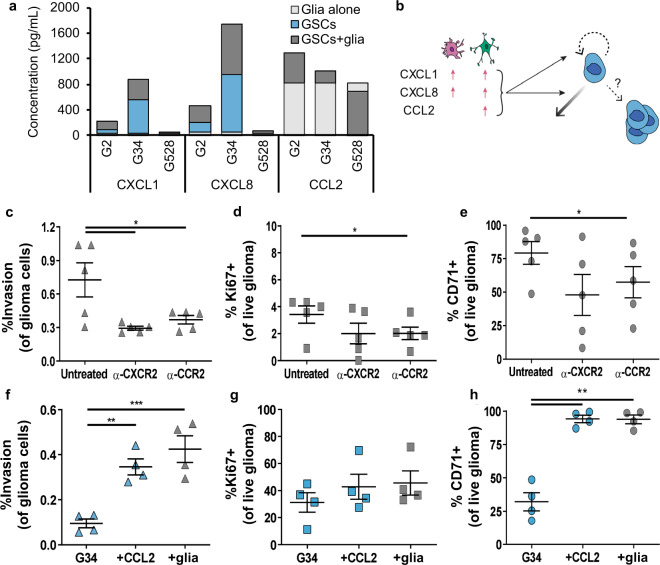
Cytokine expression is altered in tri-culture, with CCL2 able to recreate many of the TME effects on glioma outcomes. **a** Cytokine expression based on a Luminex array for hydrogel samples of astrocytes and microglia alone (AM) and in combination with GSC lines G2, G34, or G528 in static conditions. The baselines for each glia-only and GSC monoculture condition are subtracted from the respective tri-culture condition to show synergistic as opposed to additive effects. Therefore, the total height of each bar (as opposed to relative height) shows the total cytokine expression for each condition. **b** Schematic showing what is known about the three cytokines pertaining to glial expression following activation and the known effects on glioma invasion, proliferation, and stemness (counterclockwise from left). **c**–**e** Effects of blocking the cytokine receptors CXCR2 (for CXCL1 and CXCL8) and CCR2 (for CCL2) in tri-culture under flow conditions on the outcomes of invasion (**c**), proliferation (**d**), and stemness (**e**). **f**–**h** Effects of adding CCL2 to GSC monoculture hydrogels vs. the full cellular TME in static conditions for invasion (**f**), proliferation (**g**), and stemness (**h**). Statistics performed by paired *t*-tests with *p < 0.05, **p < 0.01, and ***p < 0.001.

The TME is known to cross-communicate with glioma cells to drive tumor progression^25^, and the expression levels of many different cytokines are known to vary by patient and predict overall survival. To examine glioma-glial cell communication in our model, we analyzed the cellular secretomes of three GSC lines (G2, G34, or G528) in monoculture versus in tri-culture under static (no flow) conditions. We also assessed glial cells cultured in gels alone. A 44-plex cytokine array (Luminex) revealed glial cells are a major source of pro-tumorigenic CCL2 while glioma cells express CXCL1 and CXCL8 (Fig. 4a). Prior literature suggests each cytokine can contribute to glioma cell invasion and stemness, but the effects on proliferation are less documented (Fig. 4b). These cytokines are upregulated to varying degrees in the tri-cultures, depending upon the GSC line. Glial co-culture with G2 or G34 induces upregulation of all three cytokines, but adding glia to G528 had only minor effects other than a decrease in CCL2.

To interrogate the effects of these cytokines in the glioma TME, we added blocking antibodies in the pre-solution of the tri-culture gels containing G34 (i.e., G34 + glia without flow). Blocking each cytokine directly with antibodies shows varied and unreliable results (Supplementary Fig. 8), potentially because cytokines diffuse faster than antibodies and can be sequestered by the matrix. Therefore, we used antibodies against the relevant receptors instead, namely CXCR2 for CXCL1/CXCL8 and CCR2 for CCL2. Blocking either CXCR2 or CCR2 decreased the invasion of G34 GSCs, with α-CXCR2 having a significant effect (Fig. 4c). Conversely, only blockade of CCR2 induced significant decreases in GSC proliferation and stemness (Fig. 4d, e). Furthermore, adding CCL2 into the pre-hydrogel solution with G34 alone significantly increases GSC invasion and stemness without influencing proliferation. These effects of CCL2 recreate the effects of the cellular TME on G34 (Fig. 4f–h). CCL2 is therefore playing a role in TME-driven enhancements to glioma “malignancy” and may impact individualized cancer responses.

### Cancer-associated glial activation is patient specific

**Fig. 5:**
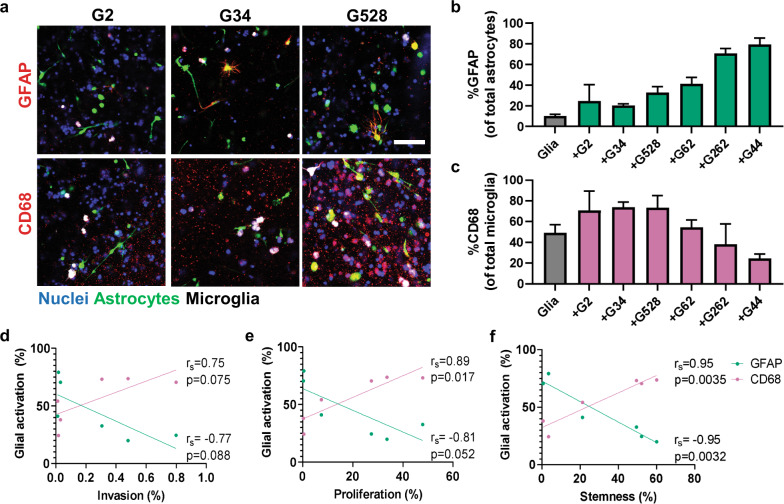
Glial reactivity to glioma cells is patient-dependent, and astrocyte reactivity correlates with glioma CD71 expression. **a** Representative immunofluorescence images showing expression of reactivity markers GFAP (red, astrocytes) or CD68 (red, microglia) for glial cells alone (AM) or in tri-culture with G2, G34, and G528. Glioma cells (blue) are labeled only with DAPI, while CellTrackers label the astrocytes (green) and microglia (white). Some G528 cells also labeled with CD68. Scale bar is 100 µm. **b** Quantified number of GFAP^+^ astrocytes as a percent of total astrocytes. **c** Quantified number of CD68^+^ microglia as a percent of total microglia. **d** Correlation plot of GFAP and CD68 expression vs. GSC invasion data. **e** Correlation plot of GFAP and CD68 expression vs. GSC Ki67 expression data. **f** Correlation plot of GFAP and CD68 expression vs. GSC CD71 expression data. Spearman correlation coefficients and *p* values are shown in respective plots, with *n* = 3 trials per cell line.

Most research into the TME focuses on how the stromal cells affect cancer. Because cancer-stromal crosstalk is bidirectional, and possibly cyclic, it equally important to determine how the cancer cells affect the stromal cells. We therefore used the TME model in static conditions (no flow) to test the effects of GSCs on glial cell phenotype. Specifically, we used immunocytochemistry to evaluate glial expression of known activation markers GFAP for astrocytes and CD68 for microglia (Fig. 5a). The baseline activation in glia-only cultures was low for astrocytes and moderate for microglia, possibly because the microglia are immortalized. The presence of GSCs influences astrocyte activation the most, with every line but G2 significantly increasing the percent of GFAP^+^ astrocytes (*p* < 0.05; Fig. 5b). The percent of CD68^+^ microglia tended to increase with G528, G2, and G34 and decreased with G262 and G44, but no statistical differences were observed with overnight culture (Fig. 5c). Unexpectedly, astrocyte activation strongly negatively correlates with microglia activation in the tri-cultures across all cell lines (Spearman *r*s = −0.9429; *p* < 0.05; Fig. 5d–f). There is also an interesting trend for the response to cluster by the original tumor subtype, but more data are necessary to support definitive conclusions.

Further analysis revealed glial activation correlates with the glioma metrics previously influenced by the cellular TME. Glioma cell invasion is strongly but not significantly correlated with either astrocyte %GFAP^+^ (Spearman *r*s = −0.767, *p* > 0.05) or microglia %CD68^+^ (*r*s = 0.747, *p* > 0.05) (Fig. 5d). While astrocyte activation also does not correlate with glioma proliferation (*r*s = −0.807, *p* > 0.05), the percent of activated microglia shows a very strong and significant correlation (*r*s = 0.892, *p* < 0.05) (Fig. 5e). Strikingly, the percentage of both activated astrocytes (*r*s = −0.953, *p* < 0.01) and microglia (*r*s = 0.951, *p* < 0.01) exhibit a very strong, significant correlation to glioma cell stemness in our model (Fig. 5f). Thus, the activation status of glial cells in the GBM TME is influenced by glioma cell phenotype. Further studies are needed to identify the signaling molecules and therapeutic implications of glial activation in GBM progression.

### In vitro viability in the TME model does not predict xenograft drug response

The ability to apply interstitial fluid flow within our model allows us to recreate a physiologically relevant mechanism of drug delivery within the TME. We tested the utility of our system for assessing tumor drug response using a selected panel of clinically relevant therapeutics. We screened the first-line treatment temozolomide and several second-line therapies commonly used in either GBM treatment or other central nervous system tumors: carboplatin, methotrexate, etoposide, irinotecan, and BCNU (carmustine). Our ultimate goal was to compare to xenograft treatments, in which examining all seven lines would be cost-prohibitive and require potentially excessive animal use. We therefore focused on comparing in vitro and in vivo responses of GSC lines G34 and G528. All in vitro drugs were applied to the gel using gravity-driven flow (~0.54 µm/s). We find the addition of the TME into the gel significantly increases the 50% inhibitory concentration (IC_50_) based on glioma cell viability (Supplementary Fig. 9). The GSCs are less responsive to almost every therapy tested in the TME condition compared to GSC monocultures in-gel alone or spheroid culture. This result is highlighted by the G34 response, which often did not reach 50% viability for any concentration tested due to high resistance conferred by the TME model.

**Fig. 6:**
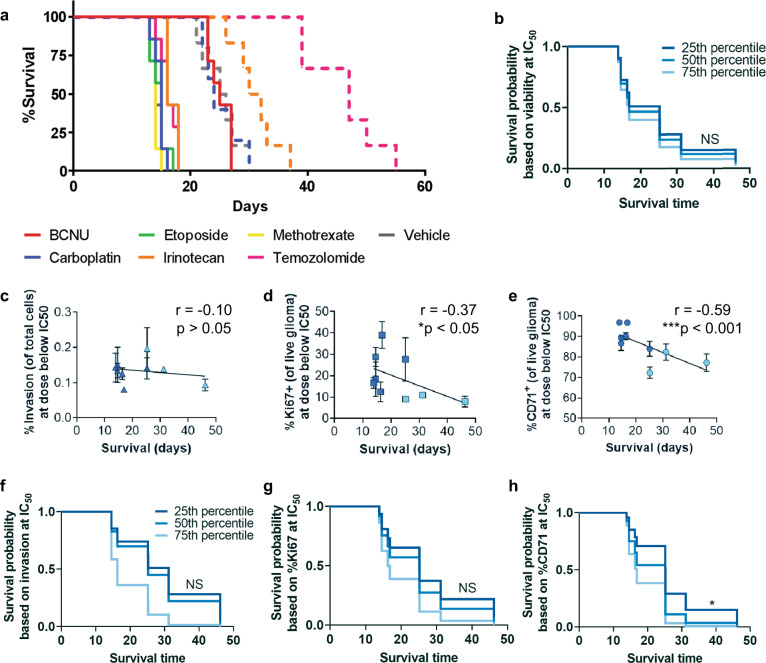
Metrics for proliferation and stemness correlate with xenograft survival, with in vitro stemness predicting in vivo drug response. **a** Kaplan–Meier plots for orthotopic xenografts of 10,000 G34 (solid lines) and 400,000 G528 (dashed lines) treated with chemotherapies at concentrations and regimens based on published literature. G528 was only tested with temozolomide, carboplatin, and irinotecan. Statistical comparisons are shown in Supplementary Fig. 10. **b** Proportional hazards model built using in vivo survival data and in vitro viability data in the TME model at the dose-below-spheroid-IC_50_ value. **c**–**e** Collective in vitro responses at the dose-below-spheroid-IC_50_ value of G34 (dark blue) and G528 (light blue) treated with drugs in the TME model for invasion (**c**), proliferation (**d**), and stemness (**e**). Each data point is *n* = 4, with 6 drug responses for G34 and 3 drug responses for G528. Pearson correlation coefficients and *p* values are shown on respective plots (*N* = 9). **f**–**h** Proportional hazards models built using in vivo survival data and data from (**c**–**e**), examining the ability to predict xenograft survival given the percent invasion (**f**), proliferation (**g**), and stemness (**h**). Only stemness was able to predict in vivo therapeutic response (by proportional hazards model with *p < 0.05). NS indicates not significant.

To determine if in vitro drug response predicts survival in mice, we implanted either GSC line G34 or G528 into NOD-SCID mice and treated animals with the same therapeutics according to informed doses and schedules are shown in Supplementary Table 4^26–29^. Fewer select drugs were used for G528, and G528-bearing mice generally have longer survival times than those with G34. Temozolomide and BCNU prolonged survival the most (Fig. 6a and Supplementary Fig. 10), although neither of these therapies had the lowest IC_50_ in any of the models we tested. We built a proportional hazards model to assess the relationship between average mouse survival time and average in vitro viability at the dose below IC_50_ (in spheroid cultures, since IC_50_s are not always reached in the TME model). Dosages used are reported in Methods. Proportional Hazards models assess the relationship between average mouse survival time and average experimental outcomes. We included averages across all the replicates within each cell line and treatment type from in vitro (Supplementary Fig. 9c–e) and in vivo (Supplementary Fig. 10) experiments. This hazard ratio model shows in vitro viability data does not predict survival of xenografted mice (Fig. 6b).

### Additional malignancy outcomes are necessary to predict drug efficacy

Given the inability of our in vitro viability dose response to predict xenograft survival, we next examined the predictive ability of the other outcomes including invasion, proliferation, and stemness. We again used the values of invasion, proliferation, and stemness at the dose below IC_50_ (from spheroids) since these metrics can decrease as a side effect of decreasing tumor cell number. (In vitro data for statistical model development are shown in Supplementary Fig. 9c–e) Correlation analysis shows invasion does not correlate with in vivo survival (*R* = 0.10; *p* > 0.5) (Fig. 6c), while both proliferation and stemness significantly negatively correlate with xenograft survival (*r* = −0.37, *p* < 0.05 and *r* = −0.59, *p* < 0.001, respectively; Fig. 6d, e). A proportional hazards model shows neither invasion (Fig. 6f) nor proliferation (Fig. 6g) predicts outcomes in mice, but the percentage of CD71^+^ cells does predict in vivo mouse survival (*p* < 0.05) (Fig. 6h). Therefore, glioma stemness in response to drug treatment, at least in our TME model, both correlates with and predicts xenograft drug response for two distinct patient-derived cell lines.

## Discussion

Advancing the fight against cancer will require the identification of new prognostic factors and potential predictors of therapeutic response. Improving the efficacy of in vitro cancer models will be an important step toward identifying more effective markers, therapies, and patient-specific drug regimens. In this regard, there is growing appreciation for the TME, both the cells and biophysical forces surrounding the tumor, and the role it plays in cancer progression and therapeutic drug response. Here, we sought to develop a model of invasive human GBM as a tunable platform in which to dissect the discrete effects of the cellular and/or biophysical elements of the brain TME. The ultimate goal is to better understand glioma cell malignancy and therapeutic response within a tissue-like environment while having control over each variable, namely the presence of astrocytes, microglia, and/or interstitial fluid flow. Future iterations of this model could include the integration of structural features (such as conduits for bulk flow like perivascular or perineural spaces), other cell types, or more complex extracellular matrix compositions.

We wanted to use physiologically relevant cellular ratios in our model and so started our design process by quantifying the cellular content of the TME in GBM patient samples. The cellular ratio, as opposed to the cellular density, was chosen because this has been directly tied to therapeutic response in models of breast cancer^4^. Analysis of adjacent sections revealed an approximately equal number of astrocytes and microglia in the TME. Previous studies have estimated twice as many astrocytes as microglia in the cortex, but it may differ in the context of cancer^30^. There are also other cell types in the TME known to influence glioma progression, including neurons^31^, oligodendrocytes^32^, and non-neural endothelial cells^33^. We chose to focus on astrocytes and microglia because these are two neural cell types previously shown individually to play major roles in GBM progression as well as therapeutic response^25,32,34,35^. In addition, although the fractional number of glial cells did not correlate with patient survival here (Supplementary Fig. 1), we previously showed the area coverage of astrocytes and microglia does significantly correlate with GBM patient survival^20^. In addition, we incorporated interstitial flow within our model given its established ability to influence GSC invasion^8^. Other models have been created to study the role of factors like the extracellular matrix or other stromal cells in GBM^36–38^, but to our knowledge, this is the first model designed for simultaneously probing the effects of three TME components (two glial cell types and interstitial flow).

The primary evaluation of the current model is based on four metrics of glioma behavior, namely invasion, proliferation, putative cancer stemness, and cell death. While stemness in cancer is widely debated, prior in silico modeling has revealed the need to effectively kill stem-like cells in breast cancer to prevent recurrence^23^. Furthermore, recent research shows glioma cells do exhibit *de facto* qualities of stemness, including the ability to differentiate into multiple functionally distinct phenotypes, which recreate a neurodevelopmental hierarchy^39^. Our patient-derived GSCs were isolated using non-adherent culture, and expression of common stemness markers like CD133—often used for GSC isolation—is not guaranteed without this positive selection step. Furthermore, expression of the purported stem cell marker CD44 is highly expressed in mesenchymal subtype GSCs but is expressed less in other subtypes, devaluing its use as a general stemness marker. Ultimately, we chose the putative stemness marker CD71 (transferrin receptor) as it is reportedly necessary for GSC maintenance^21^.

Patient-derived cells are inherently heterogeneous across and even within samples, and this patient heterogeneity was unsurprisingly a significant contributor to percent GSC invasion and proliferation in our model. Unique to our analysis, we find the addition of either glial cells or interstitial fluid flow significantly influences glioma cell expression of the putative stemness marker CD71. In addition, the presence of interstitial flow increases GSC proliferation. A connection between putative stemness and proliferation has been suggested previously^24^, and our statistical modeling and correlational analyses support the idea that invasion, proliferation, and stemness are positively co-correlated in the presence and absence of applied therapies. Nonetheless, each metric was not always significantly affected for all cell lines and conditions, supporting the idea of using multiple outcome metrics, larger sets of patient cells, and clustered data analyses to avoid missing conclusions about glioma cell responses beyond being “heterogeneous”.

Using the tunability of our system, we examined reciprocal crosstalk-based cytokine expression and glial cell activation, which may be tumor or patient specific. We identified an increase in CXCL1, CXCL8, and CCL2 upon combining glioma cells with glial cells. Glial cells appear to be the primary source of CCL2, and supplementing GSCs with CCL2 alone reproduced some effects of the TME on glioma invasion and stemness. Our analysis here only included one cell line, but previous studies found microglia secrete most of the CCL2 after being recruited by low levels of glioma-derived CCL2^40^. Importantly, CCL2 expression correlates with tumor grade^40^; is essential for recruiting regulatory immune cells into GBM tumors^41^; and can have negative implications on antiangiogenics and immunotherapies^42^. Thus, glial cells may help recapitulate an in vivo-like cytokine milieu. In addition, we find the response of glial cells to glioma cells may be patient specific. The activation status of these glial cells is strongly correlated with glioma invasion, proliferation, and stemness, though it is currently unclear which outcome drives the other or if the influence is dynamic and bidirectional. Interestingly, the glial response tends to cluster by GSC subtype (mesenchymal G2, G34, G62; classical G528; and proneural G262, G44). A prior study showed a correlation between GBM subtype and monocytic cell activation^43^, which motivates additional studies to reveal if subtype-dependent glioma-glial cell interactions exist and can influence tumor progression and/or therapy.

Toward the ultimate goal of therapeutic validation and discovery, we compared chemotherapeutic drug response in our model to that in well-established culture models. We based the initial analysis on the IC_50_ of survival, or the concentration necessary to reduce cell survival by 50%. An IC_50_ calculation requires the drug to be able to induce a near-complete response (e.g., 0% cell survival or other measurable outcomes), which was often difficult to achieve in the TME model due to increased drug resistance in the presence of the hydrogel and cellular TME. In addition, IC_50_ calculations often could not be apply to the metrics of invasion, proliferation, and stemness, since these did not consistently decrease toward zero (and often increased) at higher drug concentrations. There are also other metrics, like EC50, GI50, and GR50, with different calculation requirements and different insight about therapeutics responses^44^. It will be worth exploring the potential of these alternative metrics in future analyses within our and other model systems.

In vitro viability data are often used to screen drugs prior to pre-clinical animal testing^45^, but viability data from our in vitro model (based on %live at a dose below spheroid IC_50_) did not predict in vivo xenograft survival. There are several potential reasons why our survival data may have failed to correlate with in vivo results: Our model does not recreate the blood-brain/tumor-barrier, which impacts drug transport and provides important pro-tumor signaling. Our drug dosing concentrations and schedules, while based on prior literature, may also not capture optimal drug responses for each therapy. The xenograft model is necessarily in immunocompromised mice and therefore lacks any interactions between the tumor and a functioning adaptive immune system, though this is similar to our in vitro model. Furthermore, it may not be necessary or desirable to predict xenograft survival since these models can poorly translate to human patients despite remaining the gold standard of cancer testing^12,46^. More work is necessary to understand how best to predict outcomes in patients, but our data suggest putative markers of cancer stemness may provide important insight toward this aim. Specifically, the percent of GSCs expressing CD71 was at the dose below spheroid IC_50_ was predictive of xenograft survival across two cell lines and multiple drugs.

In summary, our goal was to develop a simplified, clinically relevant glial cell microenvironment modeling the invasive regions of GBM. We developed this model based on patient samples and cell lines and used the model to examine outcomes related to glioma cell phenotype, glioma-glial cell crosstalk, and therapeutic response. Currently, analysis in this “average” TME model only takes a few days, such that the largest hurdle would be the time required to generate patient-derived cell lines in the clinic. The model is highly tunable and may be informed by histological analysis of each patient to tune the cellular ratios or cellular identities. The model does not yet account for patient-specific ECM compositions or fluid flow rates. Histological analysis of each patient’s ECM components may add to the model but will also add time and cost, and interstitial fluid flow is only now starting to be evaluated in human GBM patients^47^. In addition, more work is required to understand which metrics are affected by interstitial fluid flow as well as the influence of flow magnitude. Ultimately, substantially more patient data and academic-clinical collaborations are needed to truly identify which model components and metrics are needed to establish transformative predictive power in glioma therapy. Our data suggest CD71 expression should be considered as the field attempts to strengthen the connection between in vitro and in vivo drug responses.

## Methods

### Study design

The foundational advancement of this study is using quantification of patient samples for the design and development of an engineered tissue model. GBM resection samples were used to develop a model of the infiltrative brain TME incorporating patient-derived glioma cells, cellular ratios representative of actual human patients, and a matrix similar in composition to the native brain ECM and the application of interstitial fluid flow. Neuropathologists and clinicians were heavily involved in the study design process, including selecting tissue samples with appropriate infiltrative areas, identifying relevant tissue areas for quantification, providing patient cells, selecting a panel of clinical drugs, and informing metric evaluation. The hydrogel material used here was based on previous studies wherein the composition was optimized to achieve relevant rates of interstitial fluid flow^8^.

### Ethics

De-identified patient samples of GBM were collected in accordance with the University of Virginia Institutional Review Board with assistance from pathologists and written informed patient consent. All procedures involving human participants (e.g., tissue collection) were conducted in accordance with the ethical standards of the same institutional review board and with the 1964 Helsinki Declaration and its later amendments or comparable ethical standards. The collection of patient-derived GSCs complied with all relevant ethical regulations, and informed consent was obtained from the patients. All animal procedures were approved by the Institutional Animal Care and Use Committees at the University of Virginia and/or Virginia Tech.

### Patient immunohistochemistry and image analysis

Patient samples are accessed through the University of Virginia Biorepository and Tissue Research Facility and selected by a neuropathologist (J.W.M.) based on a definitive diagnosis of GBM (World Health Organization grade IV). All patients had completed tumor resections at the University of Virginia between 2010 and 2013. Samples were de-identified and processed to select tumor sections that included a portion of adjacent non-bulk tumor tissue (here referred to as the infiltrative region) as identified by a neuropathologist (F.F.B.)^20^.

Formalin-fixed paraffin-embedded 8 µm sections are deparaffinized with xylene and rehydrated in graded ethanol, antigen retrieved using high pH Tris antigen unmasking solution (Vector Labs), and stained with anti-ALDH1L1 (Abcam ab56777, 1:200) and anti-Iba1 (Abcam ab5076, 1:100), followed by DAB substrate (Vector) according to manufacturer’s suggested protocols and counterstained with hematoxylin (Thermo Scientific). H&E staining was performed by the University of Virginia Biorepository and Tissue Research Facility following standard protocols. Areas at the tumor-parenchyma invasive front of tumor resections are imaged using wide-field microscopy with EVOS FL Auto (Life Technologies) and Aperio Scanscope (Leica Biosystems) and quantified using ImageJ (National Institutes of Health). Cell populations are reported as a percentage of total cells identified by the nuclear counterstain.

### Cell lines and culture

Patient-derived human GSCs were a generous gift to Benjamin Purow from Jakub Godlewski and Ichiro Nakano (who derived them while at Ohio State University). These cells (G2, G34, G44, G62, G262, G267, and G528) are maintained in suspension culture flasks in Neurobasal medium (Life Technologies) supplemented with 1% B27, 0.5% N2, 0.01% FGF, 0.1% EGF, and 0.3% L-Glutamine. Human primary cortical astrocytes are purchased from Sciencell and cultured according to the manufacturer’s suggested protocol. Human SV40-immortalized microglia are purchased from Applied Biological Materials, Inc and cultured in Dulbecco’s Modified Eagle’s Medium (Life Technologies) supplemented with 10% fetal bovine serum. All cell lines are maintained at 37 °C in a humidified incubator containing 5% CO_2_ and 21% O_2_ and tested annually for mycoplasma (negative).

### Sex profiling of patient cells

Cells are incubated with 50 ng/mL colcemid (Karyomax; Invitrogen) 4–6 h prior to fixation to enrich mitotic cells. The cells are collected and centrifuged at 1000 rpm for 5 min (used for all subsequent centrifugation steps). The cell pellet was washed once with PBS and centrifuged again. The cells are resuspended in 0.075 M KCl and incubated at 37 °C for 18 min; then 0.5 mL of freshly prepared fixative (3:1 methanol-glacial acetic acid) was added before centrifugation. The cells are resuspended in fixative added drop-wise and incubated at room temperature for 15 min before centrifugation. Cells are suspended in a final volume of 0.3–6 mL fixative (added drop-wise; final volume based on pellet size) and 12 µL is dropped onto microscope slides, which are then air-dried overnight. Human X/Y centromere enumeration probes (Metasystems Probes) are added to the sample, sealed under a coverslip with rubber cement, and placed on a hotplate at 75 °C for 3 min for probe and sample denaturation. Samples are placed in a humidified incubator at 37 °C for 4–6 h to allow probe hybridization. After removing the coverslip and any glue remnants, samples are washed in 0.4X SSC (pH 7.0) at 72 °C for 2 min and 2X SSC, 0.05% Tween-20 (pH 7.0) at room temperature for 30 s. The slides are rinsed briefly in distilled water and allowed to air dry. Antifade solution (90% glycerol and 0.5% N-propyl gallate) with 300 nM 4′,6-diamidino-2-phenylindole (DAPI) was added to the slides, sealed under a 22 × 50 mm coverslip (Corning Incorporated) with nail polish, and incubated at room temperature for 10 min prior to analysis on a Nikon Eclipse Ti inverted microscope (Nikon Instruments Inc., NY, USA) equipped with ProScan automated stage (Prior Scientific), Lumen200PRO light source (Prior Scientific), CoolSNAP HQ2 CCD camera (Photometrics), and a 60X/1.4 NA Plan-Apochromatic objective.

### CellTracker and media viability optimization

To test cellular dyes, the cells are plated on collagen-coated tissue culture plastic, fluorescently labeled with a range of concentrations of various CellTracker dyes (Life technologies) and Vybrant dyes (Life Technologies) according to the manufacturer’s suggested protocol and maintained in respective serum-free media. Cells are also tested in varying media compositions to determine optimal viability. Tested media compositions include basal astrocyte medium alone (Sciencell) or supplemented with 1% B27 without vitamin A and 0.5% N2 and/or 0.01% FGF and 0.1% EGF. For all tests, the growth of labeled cells is measured after 18, 48, and/or 72 h using the CCK-8 cell proliferation and cytotoxicity (Dojindo) kits according to manufacturers’ suggested protocols. After 72 h, cells are also assessed for viability using Live and Dead ReadyProbes Reagents (Life technologies), imaged using wide-field microscopy with EVOS FL Auto (Life technologies), and quantified using ImageJ (National Institutes of Health). Each CellTracker or Vybrant dye test was performed similarly for each glial cell type (Supplementary Fig. 3).

### Three-dimensional cell assays

Experiments are carried out in tissue culture inserts with 8 µm pore size (Sigma Aldrich CLS3374). Cells are fluorescently labeled with CellTracker dyes (Life technologies) and Vybrant dyes (Life Technologies) according to the manufacturer's suggested protocol. GBM cells (5.0 × 10^5^), astrocytes (8.0 × 10^4^), and microglia (8.0 × 10^4^) are seeded in 75 µL gel comprising (0.2% hyaluronan; ESI Bio) and 0.12% rat tail collagen I (Corning) according to cell ratios quantified from human sections. The gels are plated within 96-well tissue culture inserts with 8 µm pores (Corning) and cross-linked at 37 °C in a humidified incubator containing 5% CO_2_ and 21% O_2_. After 3 h, serum-free medium (Astrocyte Basal; Sciencell, with 1% B27, 0.5% N_2_) is added to the top and bottom of each tissue culture insert. For static conditions, the medium level is consistent inside and outside the insert, while the medium on top of the gel is greater in flow conditions. This equates to 25 µL of medium under and 125 µL of medium on top for flow, and the reverse in static. Hydrogel stiffness was previously optimized to recreate mechanical properties of the brain^22^.

### Interstitial fluid flow modeling

Fluid flow through a hyaluronan-collagen gel in a 96-well tissue culture insert was modeled using Darcy’s Law under the Fluid Flow module of COMSOL Multiphysics (Version 5.3a). All values used are shown in Supplementary Fig. 2, including some from prior literature^48–50^. The geometry for the tissue culture insert was based on manufacturer specifications, manually input into the 2D model. The height of the gel was calculated using the area of the insert membrane and the total gel volume. A custom material was applied to the domain with manually defined porosity and permeability based on the hydrogel properties. We used a finer mesh, based on the default physics. Boundary conditions were applied to each side of the geometry: wall conditions (no flow) on the left and right boundaries, and pressures prescribed at the top and bottom boundaries. The pressure head applied at the top boundary was calculated using the equation for hydrostatic pressure:

$$P = \rho \ast g \ast h$$

where *ρ* is the density of the fluid, *g* is gravity, and *h* is the height of the media on top of the gel. The height, *h*, was calculated based on the volume of medium applied to the gel and the area of the tissue culture insert. The pressure on the bottom boundary was assumed to be atmospheric. Darcy’s Law describes fluid velocity through the system as given by:

$$q = \frac{K}{{\mu \ast L}}\Delta P$$

where *q* is the Darcy flux, *K* is the permeability of the gel, *µ* is the dynamic viscosity of the fluid, *L* is the characteristic length (gel height), and Δ*P* is the pressure difference between the top and bottom of the gel. The flow velocity is calculated from Darcy flux:

$$v = \frac{q}{\varphi }$$

where $\varphi$ is the porosity of the gel. Using these governing equations, the steady-state solution for fluid velocity was calculated in COMSOL and shown in Supplementary Fig. 2.

### Invasion assay and flow cytometry

After 18 h, the gels are removed from tissue culture inserts and the membranes are cleaned using cotton swabs. Cells migrating through the porous membrane are identified by staining with DAPI (Invitrogen) and counting the number of nuclei in five representative fields per insert for three technical replicates per trial. The data are reported as the average number of cells invaded/total cells seeded × 100 (%) for each condition. In parallel, the harvested gels are digested using 0.75 mg/mL Liberase DL (Sigma Aldrich) at 37 °C for 15 min, and the cells are isolated by centrifuging for 5 min at 1100 rpm. The reisolated cells are stained using a Live/Dead dye (Life technologies, 1:750) followed by antibody staining for CD71 (eBioscience 25-0719-41, 1:100) and Ki67 (eBioscience 41-5698-82, 1:100) according to manufacturer’s suggested protocols. Flow cytometry is performed using Guava easyCyte 8HT (Millipore) with the gating strategy shown in Supplementary Fig. 4, and the data are analyzed using guavaSoft 2.7 (Millipore). In most experiments, the glioma cells are left unlabeled while the glial cells are labeled with CellTracker. The percent expression of CD71 and Ki67 in live glioma cells is therefore determined by gating on live, singlet, unlabeled glioma cells. Raw data collected is compiled in Supplementary Table 3. To compare GSC phenotypes within different model formats, marker expression is analyzed in cultures of GSCs in spheroid culture, in-gel alone, or in tri-culture gels. Analysis of tumor xenograft sections is detailed in the immunostaining section below.

### Statistical modeling of experimental outcomes

Quantile regression of the median was used to assess the relationship between experimental conditions and outcomes of interest. Experimental conditions include GSC line, addition of glial cell populations, and interstitial flow. Outcomes include invasion, proliferation, and stemness. All Interactions between experimental conditions were included in the models. Quantile regression analysis was performed using the QUANTREG procedure in SAS 9.4 (Cary, NC). A value of *p* < 0.05 was considered statistically significant (Table 1).

### Cytokine analysis

A Human XL Cytokine 44-plex array (Luminex LKTM014) was used to quantify cytokines in different cell-laden hydrogel conditions. Static hydrogel cultures (i.e., no flow) were set up as described above for conditions of GSCs only (G2, G34, or G528), GSCs plus astrocytes, and microglia, or only astrocytes and microglia (glia alone). The gels were solidified and cultured in Astrocyte basal medium +N2 +B27 without vitamin A. The gels were then transferred to a BeadBug tube on ice containing 1 mm zirconium beads (Genesee 31-212Z10) and 150 µL of T-PER™ Tissue Protein Extraction Reagent (Thermo Fisher 78510). The tubes were run on a BeadBug homogenizer twice for 30 s each to homogenize the culture samples, and the solution was pipetted up and down to ensure lysis. The solution was clarified by centrifugation at 10,000 rpm and 4 °C for 15 min, and the supernatant was collected and stored in lo-bind tubes in the −80 freezer until analysis. Protein content was analyzed using a Pierce™ Rapid Gold BCA protein assay. Selected cytokines identified by Luminex (CCL2, CXCL1, CXCL8) were validated by ELISA (R&D). The data are presented with the baselines for glia-only and G34 monoculture subtracted from the respective tri-culture condition to show synergistic as opposed to additive effects. In other words, the total height of each bar (as opposed to relative height) shows the total cytokine expression for each individual condition.

We then used our model to interrogate the role of these cytokines and therefore glioma-glial signaling in glioma cell metrics. We added either cytokine neutralizing antibodies or receptor antagonists directly into the medium and pre-hydrogel solution of G34 + glial cell cultures. Neutralizing antibodies included 0.2 µg/mL α-CXCL8 (R&D Systems MAB208), 2 µg/mL α-CCL2 (R&D Systems MAB279), and 7 µg/mL α-CXCL1 (R&D Systems MAB275). Appropriate antibody isotypes (IgG1 or IgG2b) were used as controls. Similarly, receptor antagonists were used to block signaling of CXCL1 and CXCL8 through CXCR2 (Millipore 532283, 50 nM) and CCL2 signaling through CCR2 (Millipore 227016, 10 nM). Dimethyl sulfoxide served as a vehicle control. To examine the ability of CCL2 to recreate the co-culture effects of glial cells, recombinant human CCL2 (MCP-1; Thermo Fisher, Gibco PHC1014) was added into GSC monoculture hydrogels at 10 ng/mL.

### Glial cell activation analysis

To facilitate analysis of glial cell activation, astrocytes were labeled with CellTracker Green and microglia were labeled with Vybrant DiD. The GSCs were left unlabeled. Tri-culture hydrogels were established as described above, except the gels were made as beads on parafilm until fully gelled then transferred into a 24-well plate containing 500 µL of astrocyte basal medium plus N2 and B27 without vitamin A. Following overnight culture in static conditions, the gels were fixed with 4% paraformaldehyde for 30 min at room temperature and washed three times with PBS. The samples were blocked and permeabilized using 3% goat serum and 0.03% Triton-X100 for 1 h at room temperature. The gels were stained with either rabbit anti- GFAP (Abcam ab7260, 1:1000) or rat anti-CD68 (BioLegend 137001, 1:100) in blocking buffer overnight at 4 °C. The next day, the gels were washed in PBS three times for 3 h each on a rotating shaker. Secondary antibodies were then added in the blocking buffer and incubated overnight in the fridge. Washing was repeated the following day, then the gels were counterstained with DAPI. Images were acquired on a Zeiss LSM 700 or a Zeiss LSM 880. Three images were taken at randomized locations in each gel (with gels in triplicated), and the number of marker-positive cells was counted. Data are shown as positive cells per total number of the appropriate cell type, based on CellTracker or DiD labeling of astrocytes or microglia, respectively.

### In vitro drug dosing experiments

For screening studies, 24 h after gels are seeded into transwells, a range of concentrations of BCNU, carboplatin, etoposide, methotrexate, irinotecan, and temozolomide chemotherapies are added on top of the gels (pressure head of 1 cm) to drive flow through the gels, leading to an average velocity of 0.54 µm/s. A small volume (25 µL) of chemotherapeutic-free media is added to the bottom compartment to disrupt hydrostatic pressure. After 24 h of dosing, media that flowed through the gel into the bottom compartment is carefully removed, and the same range of concentrations of each drug is added again on top to reestablish the pressure head for another 18 h. The cells are then collected for flow cytometry and the membranes fixed for invasion analysis.

### Tumor inoculation in animal studies

All animal procedures are conducted in accordance with the University of Virginia Institutional Animal Care and Use Committee (Charlottesville, VA). Eight- to ten-week-old male NOD-SCID mice are inoculated with 10,000 GSCs derived from patient G34 (*n* = 7) or 400,000 GSCs derived from patient G528 (*n* = 6) resuspended in 10 μL of neurobasal media supplemented with N2, B27 without vitamin A, and glutamax. Inoculations are performed 2 mm lateral and posterior to bregma at a depth of 2.2 mm. Seven days after inoculation, chemotherapeutics are administered intraperitoneally according to Supplementary Table 4. Animals are assessed daily for signs of distress and are euthanized accordingly when they display humane endpoint criteria.

### Immunostaining of murine tissue samples

For analyses of GSC phenotype in xenograft models, the tissue samples are collected, soaked in sucrose, cryoembedded, and sectioned at 12 μm using a Leica CM 1950. Three sections at varying depths within the tumor are immunostained with mouse anti-human nuclei (HuNu, clone 235-1, Millipore MAB1281, 1:200) followed by secondary Dylight 488 horse anti-mouse (Vector), rat Ki67 conjugated to eFluor570 (SolA15, eBioscience 41-5698-80, 1:100), and rabbit Sox2 (Millipore AB5063, 1:100) followed by secondary AlexaFluor 660 goat anti-rabbit (Life technologies). All secondary antibodies were used at 1:200. The number of invaded cancer cells was determined by first visually demarcating the tumor border based on DAPI staining (which delineates the high-density tumor bulk from the relatively lower density brain tissue) followed by counting the number of human nuclei (HuNu^+^) beyond the tumor border. Percent invasion was calculated by dividing the number of invaded cancer cells by an estimated count of total cancer cells per tissue section.

### Proportional hazards model development

Data used in this analysis includes two GSC lines (G34 and G528) and six treatments (BCNU, Carboplatin, Etoposide, Irinotecan, Methotrexate, and Temozolomide), imported and analyzed in SPSS Statistics. Measures of viability, invasion, proliferation, and stemness are measured within the in vitro TME model (*n* = 4 biological replicates with 3 technical replicates each). Specifically, we used the values of these metrics for spheroid cultures treated at the dose below cell survival IC_50_. These doses are as follows, in the same drug order as above, 100, 10, 10, 10, 10, 10 µM for G34 and 10, 10, 10, 100, 10, 10 µM for G528. Survival of mice with these cell lines and treatments was assessed, where mice exposed to the G34 cell line are treated with all six treatments (*n* = 7 mice with each treatment type), and mice exposed to the G528 cell line are treated with Carboplatin (*n* = 5 mice), Irinotecan (*n* = 6 mice), and Temozolomide (*n* = 6 mice). To assess a relationship between mouse survival and measures derived from experiments (viability, invasion, proliferation, and stemness), averages across all replicates (within cell line and treatment type) from experiments and across mice are calculated for modeling. Due to the sample size, only univariate models are considered. The hazard ratios presented indicate the change risk of death for a change of 10% of the range of variables considered. For example, viability measurements ranged from 67.44 to 96.85, a range of 29.41 units. Figures display the model predicted survival curves for a patient with low, medium, or high values for the outcomes of interest. Low, medium, and high are defined by the 25th, 50th, and 75th percentiles of the data.

### Additional statistical analysis and graphic generation

All in vitro results are repeated at least three times, and at least five animals are used for in vivo results to yield sufficient biological replicates based on power analyses. All data are presented as mean ± standard error of the mean. Independent, paired *t*-tests are used to compare all in vitro results, with analyses for invasion and cell death conducted as ratio-paired tests. Independent, unpaired *t*-tests and two-way ANOVA was used for statistical analysis of unmatched groups (in vitro glial activation and in vivo analyses). Pearson correlations were performed (except where Spearman correlation is noted) where indicated in results, and an *R* > 0.6 was considered a strong correlation, and *R* < 0.4 was considered a weak correlation, considered when *p* < 0.05. All dot plots, including Kaplan–Meyer curves, and statistical analyses are generated or performed using GraphPad Prism software, respectively. A value of *p* < 0.05 was considered statistically significant. Statistically significant differences are determined by ANOVA followed by Tukey’s *t*-tests. Illustrations were created with Adobe Illustrator, unless otherwise noted.

### Reporting summary

Further information on research design is available in the Nature Research Reporting Summary linked to this article.

## Supplementary information

Supplemental Materials

REPORTING SUMMARY

## Data Availability

The data that support the findings of this study are available from the corresponding author upon reasonable request.
