# Supplementary material for: A patient-designed tissue-engineered model of the infiltrative glioblastoma microenvironment
Source: NPJ Precis Oncol. 2022 Jul 29;6:54. doi: 10.1038/s41698-022-00290-8 (PMC9338058; doi:10.1038/s41698-022-00290-8)
Supplement: Supplementary file 1 — Supplemental Materials [file 41698_2022_290_MOESM1_ESM.pdf]

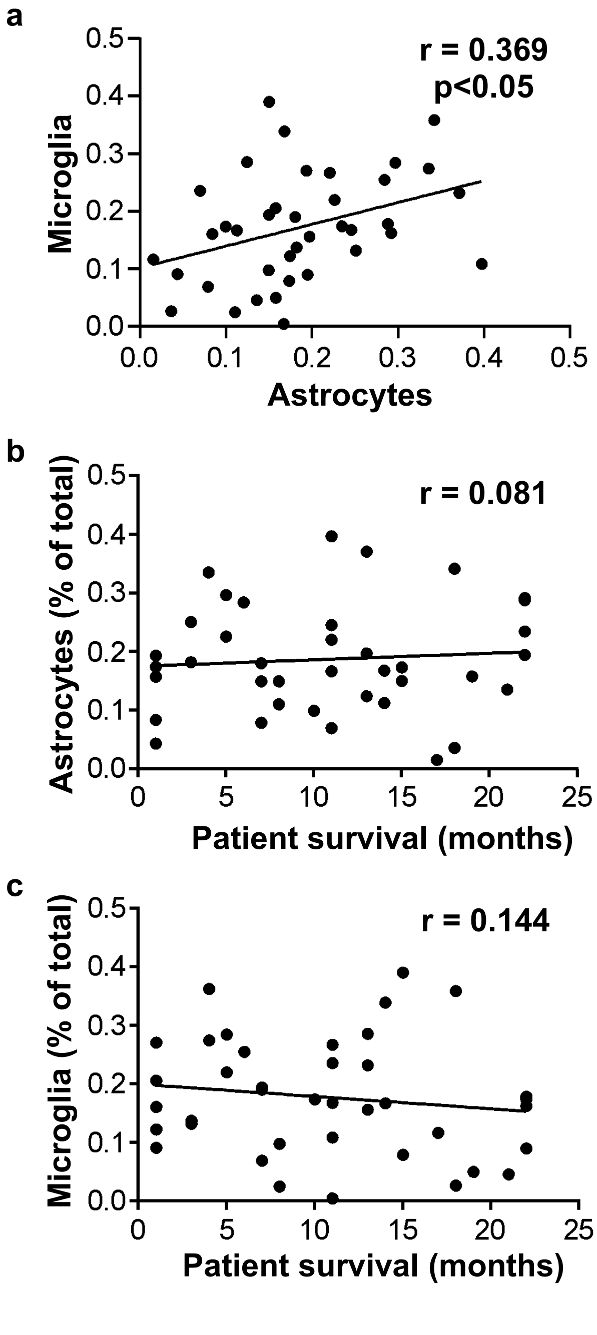

Supplementary Figure 1. Correlations of glial cell number fraction with each other and overall patient survival. A) Correlation plot showing numbers of microglia (Iba1^{+}) versus astrocytes (ALDH1L1^{+}) for each analyzed patient sample. B) Correlation plot of astrocyte number versus respective patient survival. C) Correlation plot of microglia number versus respective patient survival.

a

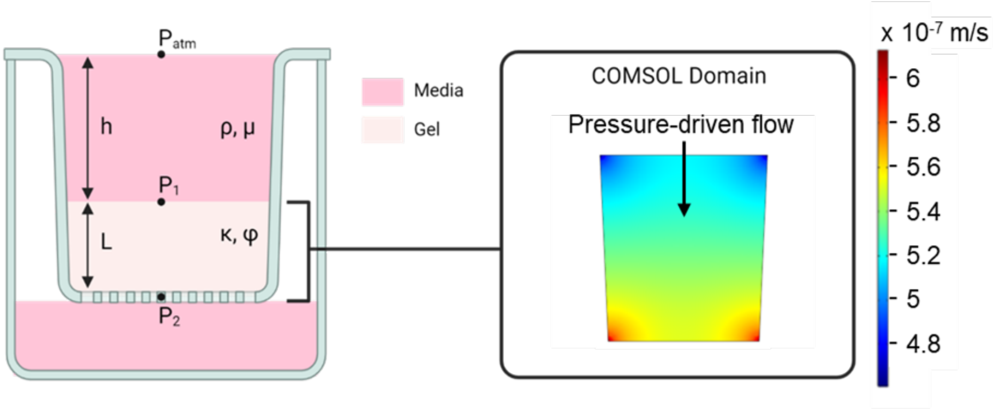

b

Parameter	Variable	Value	Unit	Source
Density of fluid	ρ	1007	kg/m^3	(*48*)
Dynamic viscosity of fluid	μ	9.79E-04	Pa·s	(*49*)
Top pressure boundary	P_1	101411	Pa	Calculated value
Bottom pressure boundary	P_2	101325	Pa	Calculated value
Porosity	φ	0.3		(*50*)
Permeability	κ	1.00E-11	cm^2	(*22*)
Height of gel	L	5.245	mm	Calculated value
Height of pressure head	h	8.71	mm	Calculated value
Gel Volume	constant	75	μL	Experimental
Media Volume	constant	125	μL	Experimental
Angle of transwell	constant	88.48	degree	Calculated value

Supplementary Figure 2. Interstitial flow modeling in the 96-well tissue culture insert. A) Schematic of the tissue culture insert, with labeled relevant parameters, and the COMSOL model of interstitial flow velocity through the hydrogel. The schematic was generated using Biorender.com. B) Table of relevant parameters used to develop the COMSOL model of interstitial flow.

a

Outcome	Cell population	Method
Viability	Live/dead$^-$	FC (%)
Proliferation	CTOrange/Live/Ki67$^+$	FC (%)
Stemness	CTOrange/Live/CD71$^+$	FC (%)
Invasion	CTOrange cells on transwell membran	Microscopy (%invasion)
Astrocyte activation	GFAP$^+$, CTGreen$^+$	ICC
Microglial activation	CD68$^+$, Vybrant DiD$^+$	ICC

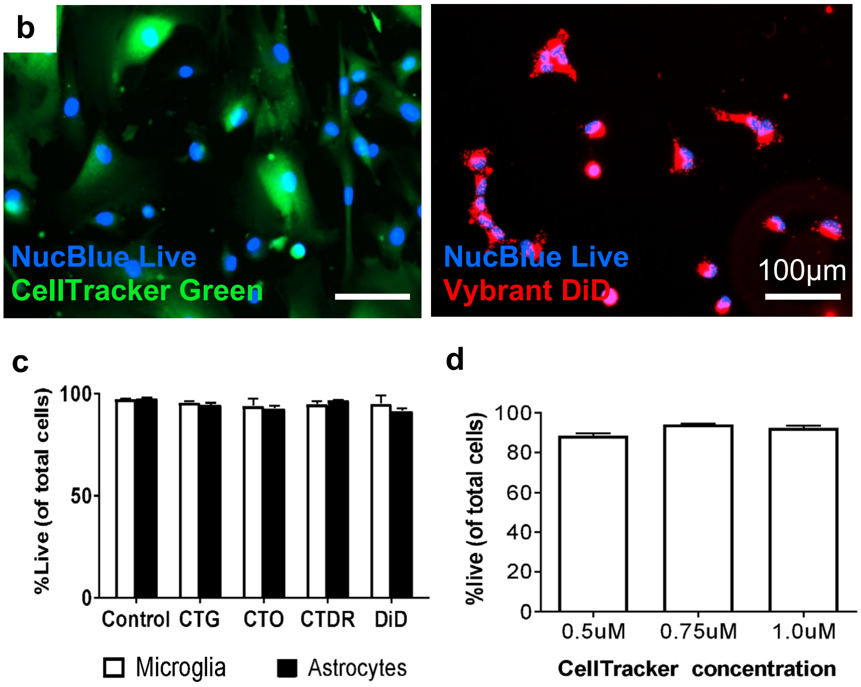

Supplementary Figure 3. Metric summary and cell dye optimization for the infiltrative human glioblastoma TME model. A) Summary of outcomes and metrics used to evaluate the infiltrative TME model. C) Representative fluorescence images of human astrocytes labeled with CellTracker Green (left) and human microglia labeled with Vybrant DiD (right). D) Glial cell survival following labeling with various cell dyes. E) Glial cell survival with different concentrations of CellTracker dye.

`

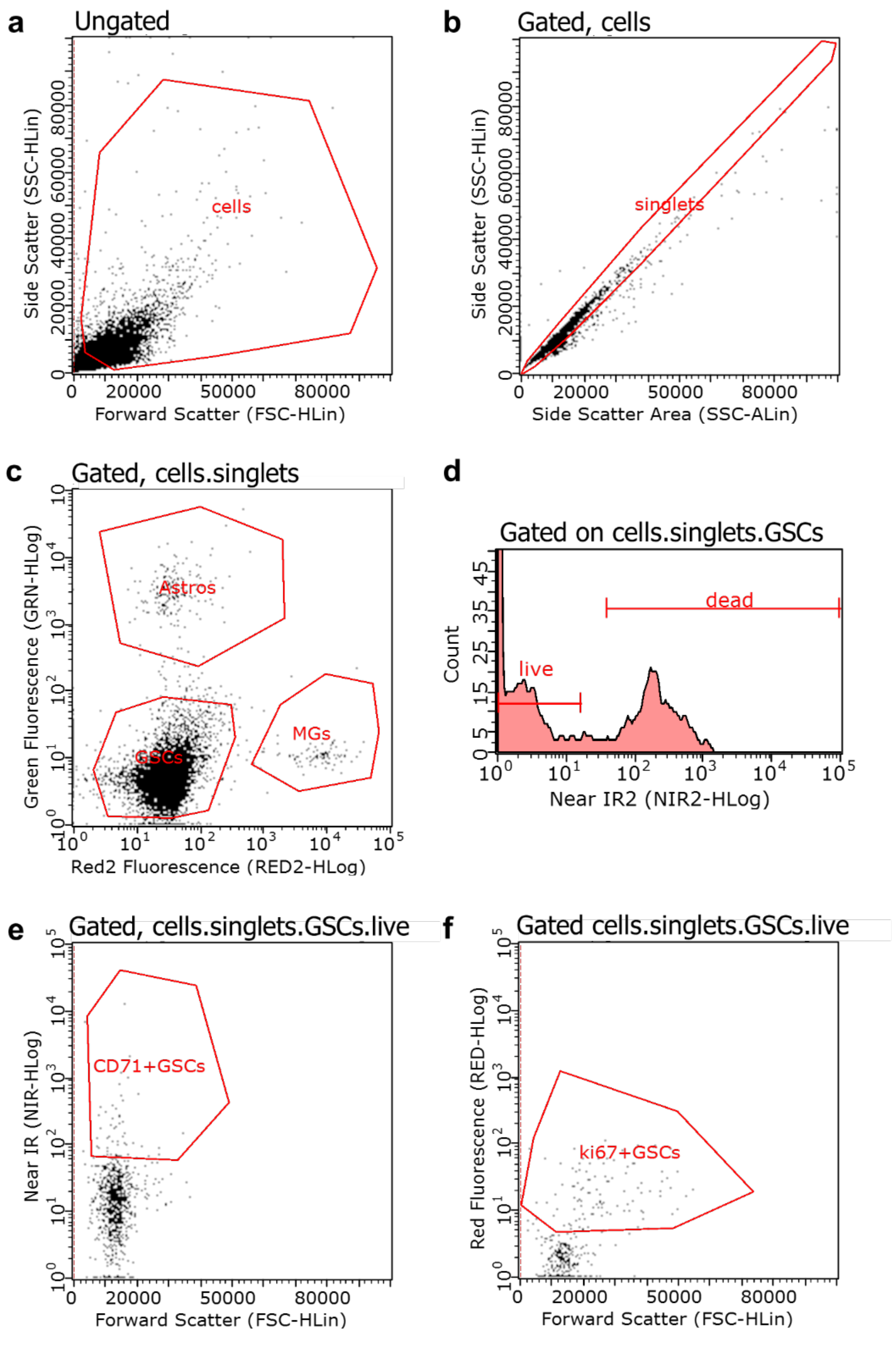

Supplementary Figure 4: Gating strategy for TME model. A) FSC/SSC plot for identification of cells. B) Singlet gating C) Identification of cellular subsets by CellTracker. D) Viability of glioma stem cells. E) CD71+ live glioma cells. F) Ki67+live glioma cells. Plots shown are from G528 cells in the TME model represented in Figure 3. Figures generated using Millipore InCyte 3.3.

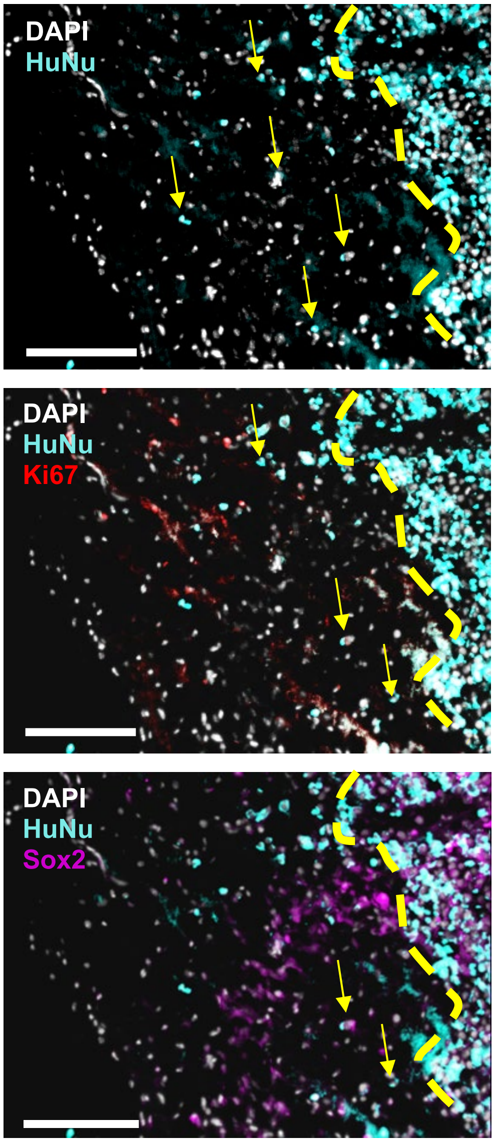

Supplementary Figure 5. Tumor cell markers in an orthotopic xenograft model within regions of invasion. Representative fluorescence images of G34 tumor xenografts related to data in Figure 2. The tumor border was demarcated based on human nuclear antigen staining (HuNu; top panel). The number of invaded tumor cells staining positive for Ki67 (proliferation; middle panel) or Sox2 (stemness; bottom panel) are counted and reported as a percent of invaded cells. Arrows indicate representative positively-stained cells.

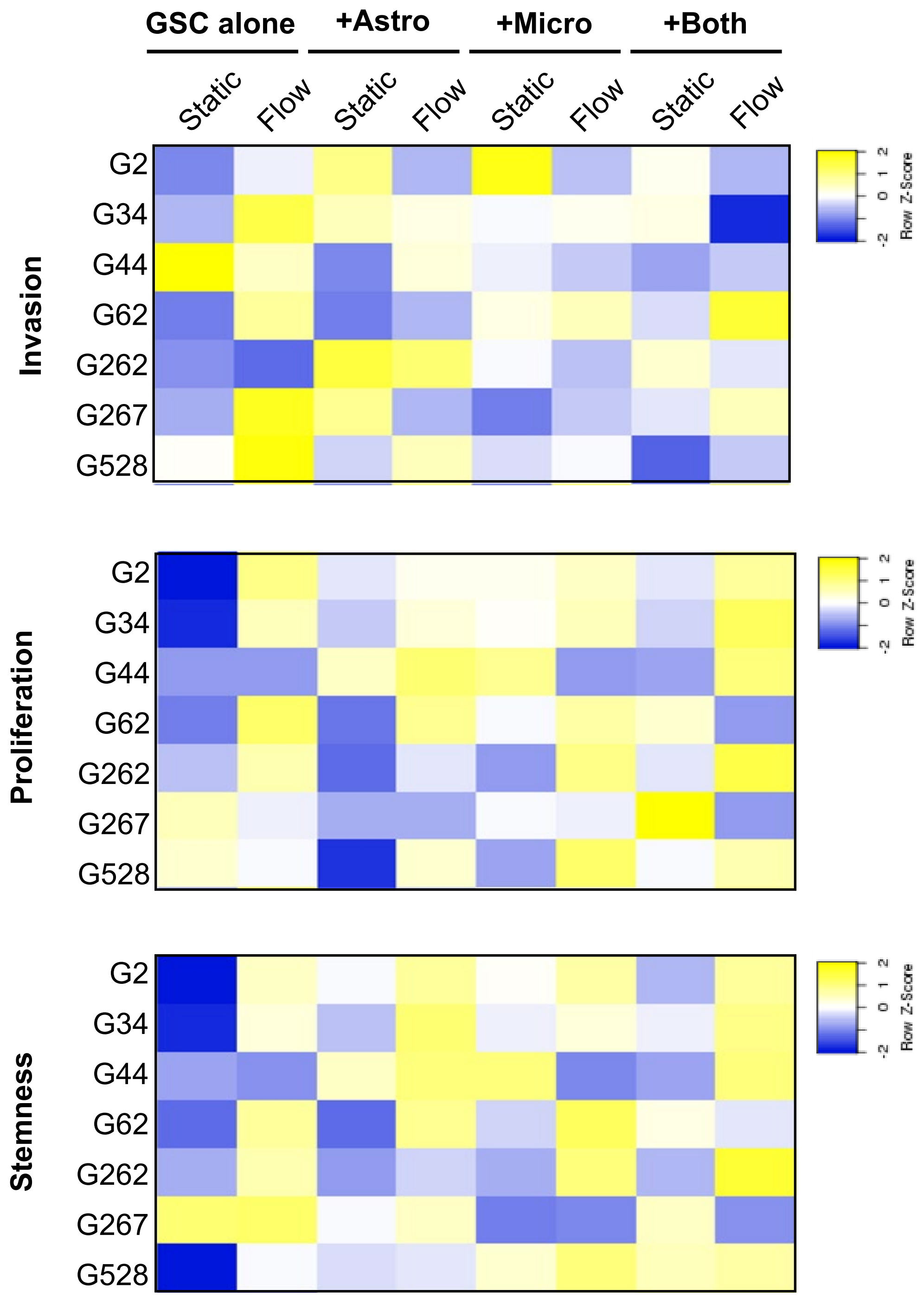

Supplementary Figure 6. Heatmap representations of changes in glioma cell metrics (invasion, proliferation, and stemness) in response to each combination of TME elements. Data are represented on a normalized z-score from -2 (blue) to +2 (yellow).

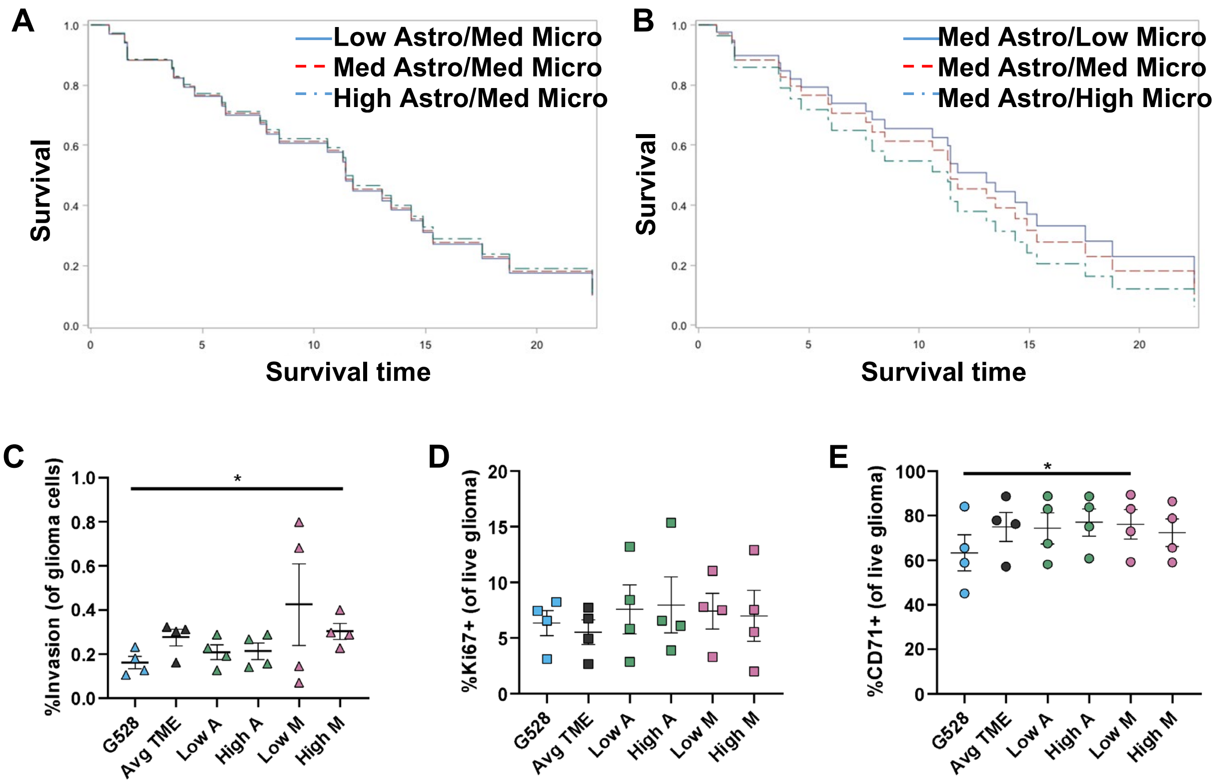

Supplementary Figure 7. Ratiometric tuning of glial cell numbers influences glioma cell outcomes. Analyzed patient samples were grouped based on the numbers of astrocytes and microglia quantified in infiltrative regions, and proportional hazards models were built to predict patient survival based on TME composition. Resulting Kaplan-Meier curves for varied astrocyte numbers (A) and microglia numbers (B) show microglia have the strongest effect on overall survival. In our TME model, we varied our standard glial ratio (1:1) by 25% to represent high or low ratios (e.g., 0.75:1 for low astrocytes and medium microglia), and examined the effects on glioma cell invasion (C), proliferation (D), and stemness (E).

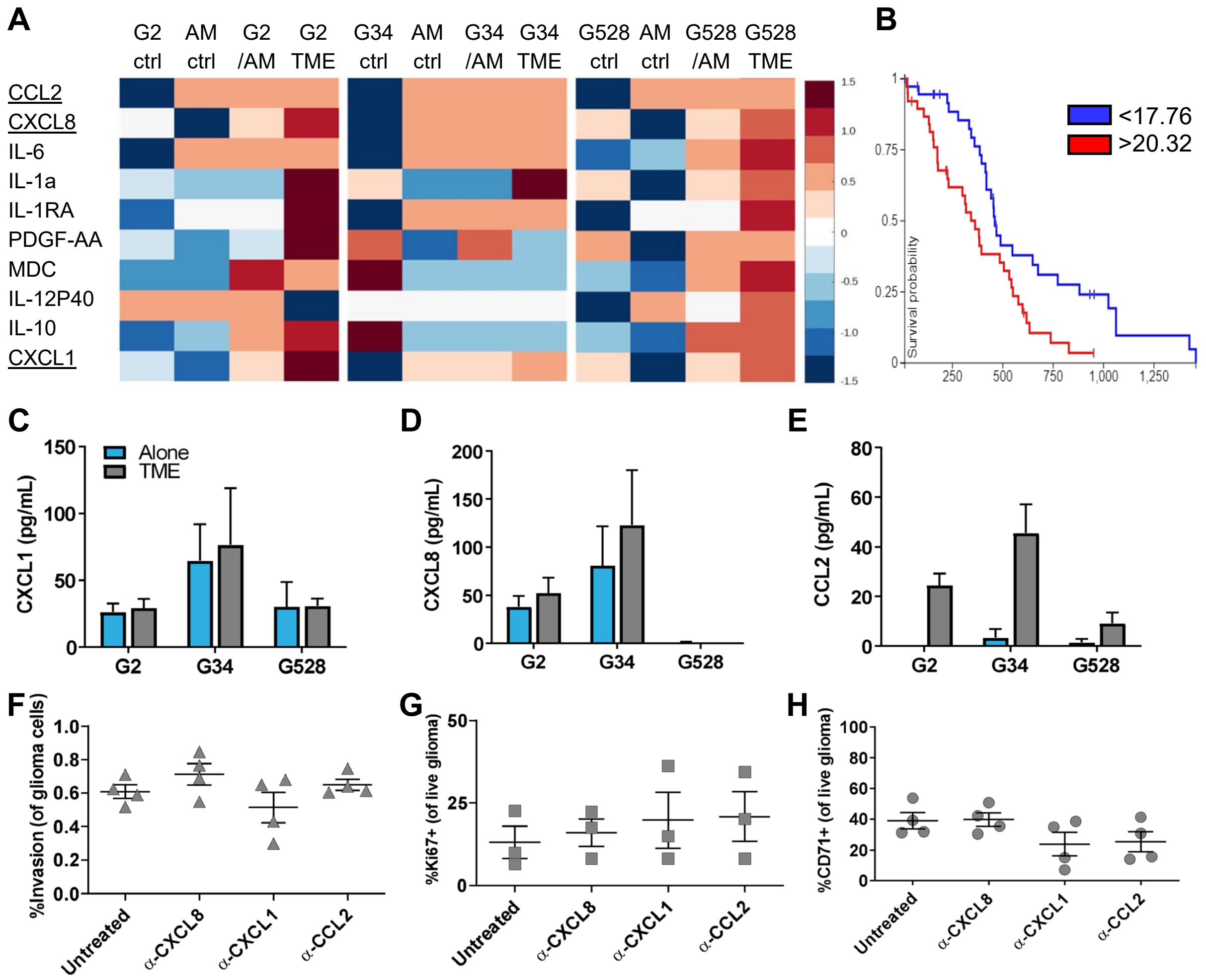

Supplementary Figure 8. Cytokine contributions from the cellular TME. A) Heatmap of cytokine data obtained using a Luminex array. CCL2 (MCP1), CXCL8 (IL8), and CCL1 (GRO1) were further explored based on high expression in the GSC+TME conditions. B) Survival data from The Cancer Genome Atlas for CCL2, showing survival of patients in the highest and lowest quartiles of CCL2 expression ($p<0.01$; $n=38$). C-E) Quantification of cytokines in tri-culture by ELISA for CXCL1 (C), CXCL8 (D), and CCL2 (E). (F-H) Blocking studies performed using antibodies against the identified cytokines and subsequent assessment of glioma cell invasion (F), proliferation (G), and stemness (H). Cytokine blocking showed minimal effect on all metrics in contrast to receptor blocking, possibly due to limitations in antibody transport (vs. cytokines) or concentration effects.

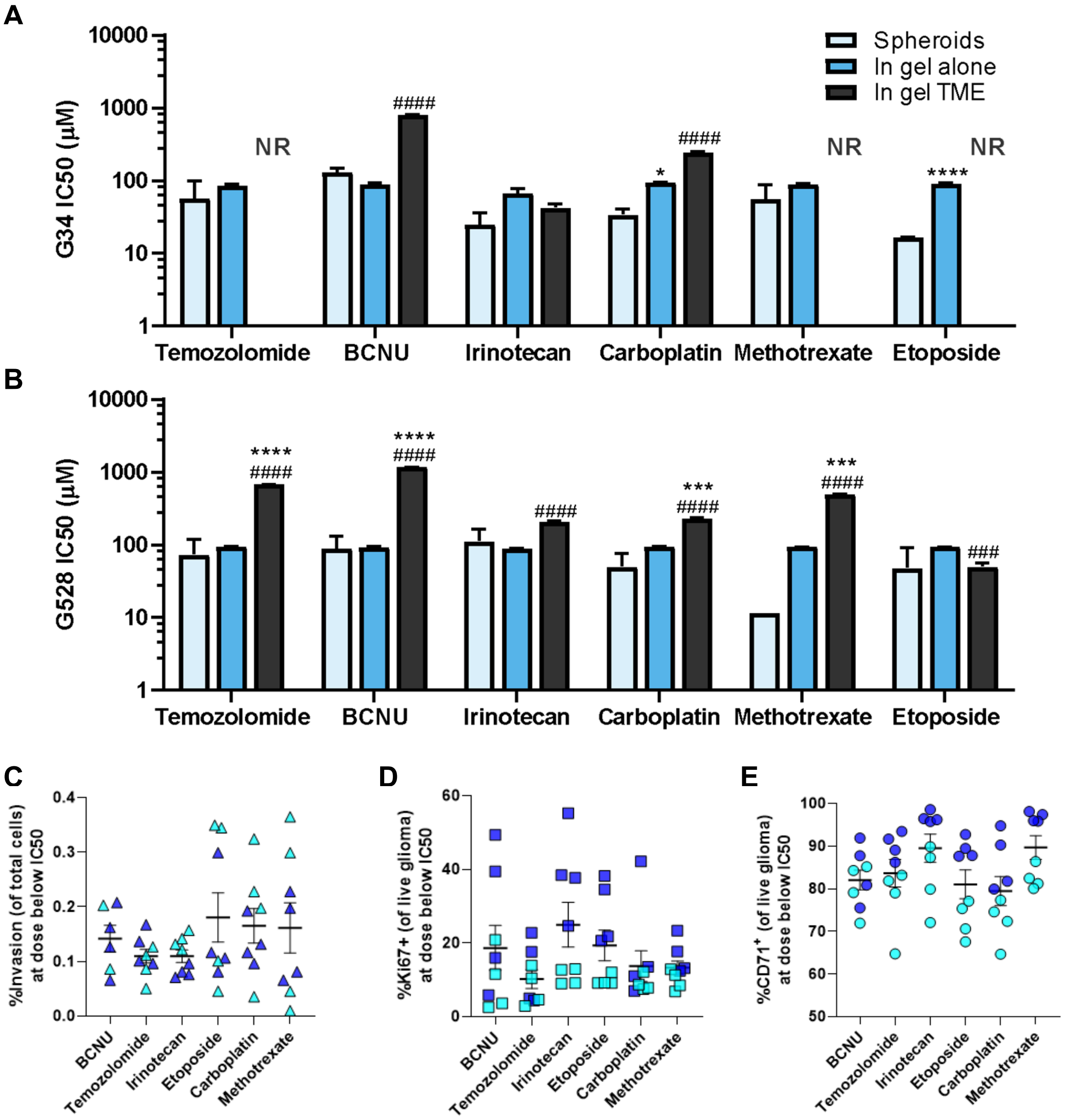

Supplementary Figure 9. Drug response *in vitro* data. IC_{50} data based on cell survival for six different drugs with G34 (A) and G528 (B) cells cultured either as spheroids, in 3D hydrogels alone, or in 3D hydrogel tri-cultures with glial cells. NR = IC_{50} concentration Not Reached up to 1000 μM. Data are shown on log scale. Statistics conducted using paired t-tests. Asterisks (*) show comparisons to spheroids data and pound symbols (#) show comparisons to 'in gel alone' data, with *p<0.05, ***p<0.001, and ****p<0.0001. (C-E) Glioma cell invasion (C), proliferation (D), and stemness (E) in the tri-culture TME model at the dose below IC_{50} (based on treatment in spheroids). G34 are shown in light blue, and G528 are shown in dark blue. This data was used to generate the proportional hazards model prediction of xenograft survival in **Figure 6**

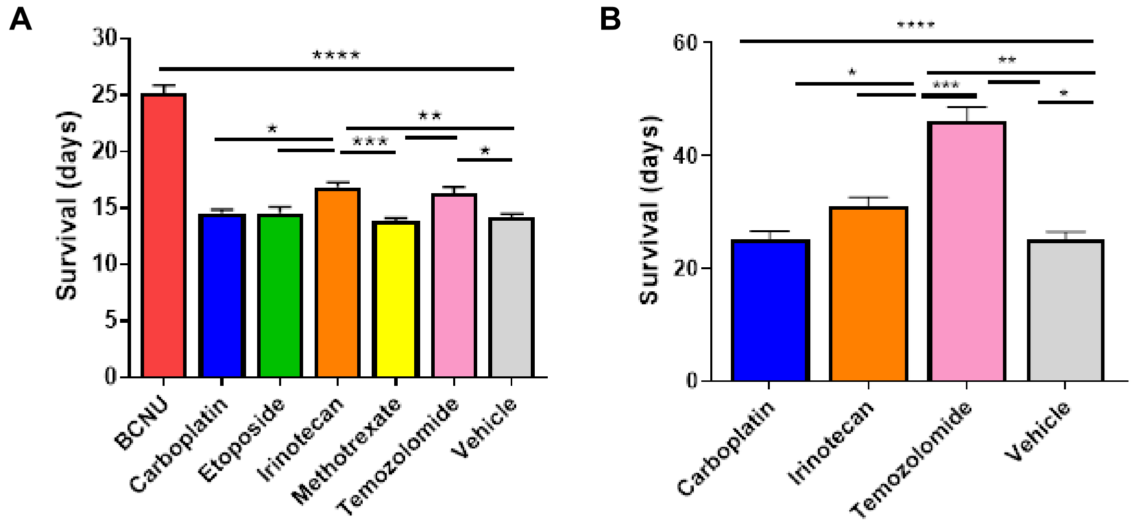

Supplementary Figure 10. Xenograft drug survival data. Average survival times and statistics for orthotopic xenografts of G34 (A) and G528 (B) in NOD-SCID mice treated with chemotherapeutics according to **Table S2**. Each group comparison was conducted using an unpaired t-test, with *p<0.05, **p<0.01, ***p<0.001, and ****p<0.0001.

Supplementary Table 1. Histological patient sample data analysis.

Sample	Astrocyte Ratio	Microglia Ratio	Mucin Ratio
GBM-4	0.1949891068	0.09041394336	0.62569
GBM-6 2A	0.1891031656	0.1928350321	0.64789
GBM-11	0.1357409714	0.04607721046	0.79901
GBM-12 2A	0.2822304493	0.05683084354	0.480625
GBM-17 2A	0.04358208955	0.09134328358	0.43859
GBM-20 2A	0.1104166667	0.025	0.84488
GBM-24 2C	0.2874119106	0.3625498008	-
GBM-27 1A	0.3711587213	0.2324050897	0.46837
GBM-29 3A	0.1145005867	0.1318380036	0.795895
GBM-35 4A	0.2207725498	0.2671514612	-
GBM-42 3A	0.2509529776	0.1322360099	-
GBM-49 2A	0.2614579345	0.2524955495	0.554915
GBM-52 5A	0.1577181208	0.2060092892	0.67817
GBM-53 1A	0.1821699926	0.1376145547	0.49555
GBM-62 2B	0.09950463525	0.1739769371	0.45681
GBM-1	0.1804949054	0.1906841339	0.39123
GBM-13	0.158	0.05	0.36316
GBM-19 2B	0.06984478936	0.2361419069	0.51042
GBM-22 2D	0.1125	0.1673913043	0.64496
GBM-23 2A	0.01548672566	0.1167035398	0.47689
GBM-25 2C	0.1734496124	0.07945736434	0.62668
GBM-30	0.2844035874	0.255169652	0.50452
GBM-41 2B	0.1680233325	0.339396619	0.51113
GBM-47	0.1243213742	0.2858774502	0.60358
GBM-50 3C	0.1502595866	0.3907329569	0.67009
GBM-51	0.2457187634	0.1681016853	0.78559
GBM-57 2A	0.1495834468	0.09807820213	0.56544
GBM-58 1A	0.1839453043	0.1967514431	0.74624
GBM-59 1A	0.3358298042	0.2749147042	-
GBM-60 2A	0.08392582671	0.1612140624	0.67457
GBM-61 2B	0.1971108294	0.1562561739	0.53584
GBM-63 1A	0.2716934849	0.1719340636	0.49094333
GBM-43 1A	0.1433172836	0.2858774502	-

Supplementary Table 2. Characteristics of patient-derived glioma stem cells.

GSC	Sex	Subtype	IDH
G2	Female	Mesenchymal	WT
G34	Female	Mesenchymal	WT
G44	Male	Proneural	WT
G62	Female	Mesenchymal	WT
G262	Male	Proneural	WT
G267	Female	Mesenchymal	WT
G528	Female	Classical	WT

Supplementary Table 3. In vitro TME model data combined.

			Invasion (% of total cells)			Stemness (%CD71)			Proliferation (% Ki67)		
Glioma Stem Cell Line	Cell Mixture	Fluid Condition	Average	Standard Deviation	N	Average	Standard Deviation	N	Average	Standard Deviation	N
G2	G2 only	Static	0.532	0.169	4	18.012	5.344	3	7.594	4.627	3
		Flow	0.737	0.208	4	63.745	27.762	4	56.600	2.152	3
	G2 + A	Static	0.754	0.237	4	55.116	19.941	3	24.005	13.591	3
		Flow	0.604	0.212	4	70.056	19.021	4	39.181	1.399	3
	G2 + M	Static	1.110	0.939	4	69.432	11.551	3	37.050	4.194	3
		Flow	0.713	0.334	4	69.583	21.429	4	46.251	9.131	3
	G2 + AM	Static	0.738	0.282	4	52.138	7.512	3	27.402	3.098	3
		Flow	0.901	0.660	4	70.142	25.094	4	48.569	15.849	3
G34	G34 only	Static	0.355	0.174	3	16.627	10.030	4	14.570	8.306	4
		Flow	0.527	0.019	3	56.398	35.074	4	56.789	8.501	3
	G34 + A	Static	0.422	0.245	4	40.991	15.652	4	30.923	4.139	4
		Flow	0.424	0.219	4	71.763	12.071	4	39.503	16.627	4
	G34 + M	Static	0.616	0.331	4	48.692	23.056	4	36.387	1.732	4
		Flow	0.586	0.322	4	57.115	26.104	4	42.805	6.847	4
	G34 + AM	Static	0.532	0.186	4	49.194	19.006	4	32.062	3.713	4
		Flow	0.340	0.150	4	68.496	21.879	4	51.311	9.396	4
G44	G44 only	Static	0.049	0.012	3	0.010	1.710	3	0.421	0.277	3
		Flow	0.031	0.013	3	2.767	1.895	3	0.356	0.216	3
	G44 + A	Static	0.014	0.005	3	8.825	4.725	3	1.422	0.515	3
		Flow	0.029	0.004	3	11.230	7.234	3	1.947	1.427	3
	G44 + M	Static	0.025	0.019	3	11.176	10.087	3	1.739	1.665	3

		Flow	0.021	0.005	3	2.308	0.548	3	0.380	0.160	3
	G44 + AM	Static	0.017	0.006	3	3.490	2.165	3	0.453	0.194	3
		Flow	0.020	0.009	3	11.246	9.320	3	1.853	1.463	3
G62	G62 only	Static	0.010	0.004	3	8.092	3.583	3	3.312	2.696	3
		Flow	0.016	0.008	3	25.721	15.552	3	9.747	11.985	3
	G62 + A	Static	0.010	0.001	3	8.450	3.285	3	3.154	2.001	3
		Flow	0.011	0.006	3	26.325	8.161	3	8.820	7.805	3
	G62 + M	Static	0.014	0.008	3	15.872	9.983	3	6.391	4.108	3
		Flow	0.015	0.004	3	29.695	15.301	3	8.355	9.785	3
	G62 + AM	Static	0.012	0.006	3	21.205	11.209	3	7.442	2.256	3
		Flow	0.018	0.007	3	17.264	4.338	3	4.020	2.889	3
G262	G262 only	Static	0.013	0.009	3	0.506	0.542	3	0.229	0.162	3
		Flow	0.009	0.009	3	2.008	1.072	3	0.396	0.195	3
	G262 + A	Static	0.046	0.024	3	0.259	0.244	3	0.125	0.027	3
		Flow	0.040	0.004	3	0.830	0.326	3	0.280	0.133	3
	G262 + M	Static	0.025	0.001	3	0.535	0.116	3	0.185	0.090	3
		Flow	0.019	0.006	3	2.480	2.083	3	0.460	0.131	3
	G262 + AM	Static	0.031	0.014	3	0.619	0.289	3	0.281	0.109	3
		Flow	0.023	0.002	3	3.130	0.436	3	0.527	0.264	3
G267	G267 only	Static	0.023	0.003	3	21.072	10.103	3	2.288	0.939	3
		Flow	0.035	0.011	3	21.453	7.003	3	1.868	1.135	3
	G267 + A	Static	0.030	0.001	3	16.641	5.581	3	1.495	0.802	3
		Flow	0.023	0.003	3	18.693	1.543	3	1.463	0.984	3
	G267 + M	Static	0.021	0.008	3	12.016	4.357	3	1.895	0.498	3
		Flow	0.024	0.004	3	12.529	6.662	3	1.878	2.080	3

	G267 + AM	Static	0.025	0.003	3	18.445	6.279	3	3.264	1.884	3
		Flow	0.029	0.005	3	12.740	3.240	3	1.387	1.124	3
G528	G528 only	Static	0.507	0.104	3	26.299	18.542	3	67.036	13.388	3
		Flow	0.787	0.404	3	64.302	28.938	3	49.539	23.573	4
	G528 + A	Static	0.451	0.058	3	59.893	14.546	3	41.498	21.423	4
		Flow	0.586	0.192	3	47.239	26.447	4	51.414	10.896	4
	G528 + M	Static	0.458	0.103	3	54.511	13.538	4	46.053	11.780	4
		Flow	0.493	0.232	3	64.022	27.981	4	55.010	10.938	4
	G528 + AM	Static	0.306	0.070	3	57.797	21.203	4	49.476	8.454	4
		Flow	0.441	0.107	3	59.504	33.771	4	52.396	16.818	4

Supplementary Table 4. ***In vivo*** **drug dosing paradigm.**

Drug	Dose (mg/kg)	Concentration (mg/ml)	Schedule (days administered)	Reference
Temozolomide	5	1	7,8,9,10,11	(*23*), (*27*)
BCNU (carmustine)	25	5	7, 10	(*26*)
Carboplatin	10	2	7, 10	(*29*)
Etoposide	3	0.6	7,8,9,10,11	(*24*)
Irinotecan	4	0.8	7,8,9,10,11	(*26*)
Methotrexate	25	5	7, 10	(*29*)
Vehicle (10% DMSO in saline)	5	1	7, 10	
