# Supplementary material for: A patient-designed tissue-engineered model of the infiltrative glioblastoma microenvironment
Source: NPJ Precis Oncol. 2022 Jul 29;6:54. doi: 10.1038/s41698-022-00290-8 (PMC9338058; doi:10.1038/s41698-022-00290-8)
Supplement: Supplementary file 2 — REPORTING SUMMARY [file 41698_2022_290_MOESM2_ESM.pdf]

nature portfolio

Corresponding author(s): JM Munson

Last updated by author(s): Jan 20, 2022

Reporting Summary

Nature Portfolio wishes to improve the reproducibility of the work that we publish. This form provides structure for consistency and transparency in reporting. For further information on Nature Portfolio policies, see our Editorial Policies and the Editorial Policy Checklist.

Statistics

For all statistical analyses, confirm that the following items are present in the figure legend, table legend, main text, or Methods section.

n/a	Confirmed	
☐	☒	The exact sample size (n) for each experimental group/condition, given as a discrete number and unit of measurement
☐	☒	A statement on whether measurements were taken from distinct samples or whether the same sample was measured repeatedly
☐	☒	The statistical test(s) used AND whether they are one- or two-sided<br>*Only common tests should be described solely by name; describe more complex techniques in the Methods section.*
☐	☒	A description of all covariates tested
☐	☒	A description of any assumptions or corrections, such as tests of normality and adjustment for multiple comparisons
☐	☒	A full description of the statistical parameters including central tendency (e.g. means) or other basic estimates (e.g. regression coefficient) AND variation (e.g. standard deviation) or associated estimates of uncertainty (e.g. confidence intervals)
☐	☒	For null hypothesis testing, the test statistic (e.g. F, t, r) with confidence intervals, effect sizes, degrees of freedom and P value noted<br>*Give P values as exact values whenever suitable.*
☒	☐	For Bayesian analysis, information on the choice of priors and Markov chain Monte Carlo settings
☒	☐	For hierarchical and complex designs, identification of the appropriate level for tests and full reporting of outcomes
☐	☒	Estimates of effect sizes (e.g. Cohen's d, Pearson's r), indicating how they were calculated

Our web collection on statistics for biologists contains articles on many of the points above.

Software and code

Policy information about availability of computer code

Data collection	guavaSoft 2.7; Zeiss Zen 3.4; EVOS FL Auto 2 Software; Luminex xPONENT software; Aperio Scanscope software;
Data analysis	JMP 16; Graphpad Prism software

For manuscripts utilizing custom algorithms or software that are central to the research but not yet described in published literature, software must be made available to editors and reviewers. We strongly encourage code deposition in a community repository (e.g. GitHub). See the Nature Portfolio guidelines for submitting code & software for further information.

Data

Policy information about availability of data

All manuscripts must include a data availability statement. This statement should provide the following information, where applicable:

- Accession codes, unique identifiers, or web links for publicly available datasets
- A description of any restrictions on data availability
- For clinical datasets or third party data, please ensure that the statement adheres to our policy

The data that support the findings of this study are available from the corresponding author upon reasonable request.

Field-specific reporting

Please select the one below that is the best fit for your research. If you are not sure, read the appropriate sections before making your selection.

☒ Life sciences ☐ Behavioural & social sciences ☐ Ecological, evolutionary & environmental sciences

For a reference copy of the document with all sections, see nature.com/documents/nr-reporting-summary-flat.pdf

Life sciences study design

All studies must disclose on these points even when the disclosure is negative.

Sample size	All in vitro results are repeated at least three times, and at least five animals are used for in vivo results to yield sufficient biological replicates based on power analyses
Data exclusions	No data were excluded
Replication	Some analyses, e.g., invasion and glial cell activation, were occasionally analyzed by more than one independent scientist to ensure reproducibility
Randomization	Animals in the xenograft survival study were randomly assigned to a treatment group on the first day of treatment
Blinding	In vitro data were collected and analyzed by well, later unblinded and averaged into experimental groups. Animals in the xenograft study were assigned numbers, such that only the person giving treatment on a given day knew which group to which those animals belonged

Reporting for specific materials, systems and methods

We require information from authors about some types of materials, experimental systems and methods used in many studies. Here, indicate whether each material, system or method listed is relevant to your study. If you are not sure if a list item applies to your research, read the appropriate section before selecting a response.

Materials & experimental systems

n/a	Involved in the study	
☐	☒	Antibodies
☐	☒	Eukaryotic cell lines
☒	☐	Palaeontology and archaeology
☐	☒	Animals and other organisms
☐	☒	Human research participants
☒	☐	Clinical data
☒	☐	Dual use research of concern

Methods

n/a	Involved in the study	
☒	☐	ChIP-seq
☐	☒	Flow cytometry
☒	☐	MRI-based neuroimaging

Antibodies

Antibodies used	mouse anti-human nuclei (HuNu, clone 235-1, Millipore); rat Ki67 conjugated to eFluor570 (SolA15, eBioscience); rabbit Sox2 (Millipore); rabbit anti- GFAP (Abcam ab7260); rat anti-CD68 (BioLegend 137001); mouse CD71 (eBioscience); rat anti-Ki-67 (eBioscience); mouse anti-ALDH1L1 (Abcam); goat anti-Iba1 (Abcam); secondary antibodies were donkey anti-mouse/rabbit/goat/rat
Validation	Each antibody validation statement can be found on the respective manufacturer's website

Eukaryotic cell lines

Policy information about cell lines

Cell line source(s)	Human astrocytes (Sciencell); human microglia (Applied Biological Materials Inc); Patient-derived human glioblastoma stem cells (GSCs) were a generous gift to Benjamin Purow from Jakub Godlewski and Ichiro Nakano (who derived them while at Ohio State University).
Authentication	No authentication was performed for the commercial cell lines. The patient-derived cell lines were regularly submitted for RPPR analysis to authenticate their purity and subtype identity
Mycoplasma contamination	All cell lines were tested negative for mycoplasma infection
Commonly misidentified lines (See ICLAC register)	*Name any commonly misidentified cell lines used in the study and provide a rationale for their use.*

Animals and other organisms

Policy information about studies involving animals; ARRIVE guidelines recommended for reporting animal research

Laboratory animals	8–10 week old male NOD-SCID mice
Wild animals	N/A
Field-collected samples	N/A
Ethics oversight	All animal procedures were approved by the Institutional Animal Care and Use Committees at the University of Virginia and/or Virginia Tech.

Note that full information on the approval of the study protocol must also be provided in the manuscript.

Human research participants

Policy information about studies involving human research participants

Population characteristics	Sex 54% male<br>Median age at diagnosis 62.5 years<br>Median survival 11 months<br>MGMT hypermethylation 29%<br>IDH1-positive 9%<br>Region of tumor 19% Frontal, 52% Temporal, 14% Parietal, 14% Occipital
Recruitment	Histological samples were archived at the University of Virginia with patient consent
Ethics oversight	De-identified patient samples of glioblastoma were collected in accordance with the University of Virginia Institutional Review Board with assistance from pathologists. All procedures involving human participants (e.g., tissue collection) were conducted in accordance with the ethical standards of the same institutional review board and with the 1964 Helsinki declaration and its later amendments or comparable ethical standards.

Note that full information on the approval of the study protocol must also be provided in the manuscript.

Flow Cytometry

Plots

Confirm that:

- [x] The axis labels state the marker and fluorochrome used (e.g. CD4-FITC).
- [x] The axis scales are clearly visible. Include numbers along axes only for bottom left plot of group (a 'group' is an analysis of identical markers).
- [ ] All plots are contour plots with outliers or pseudocolor plots.
- [x] A numerical value for number of cells or percentage (with statistics) is provided.

Methodology

Sample preparation	Cell-laden hydrogels were digested using 0.75 mg/mL Liberase DL (Sigma Aldrich) at 37? for 15 minutes, and the cells are isolated by centrifuging for 5 mins at 1100 rpm. The reisolated cells were blocked using 10% FBS for 15 minutes on ice, then washed with PBS. Cells were stained using a Live/Dead dye (Life technologies) for 15 minutes and washed twice with PBS. Extracellular labeling was performed by antibody staining for CD71 (eBioscience) in flow buffer, for 15 minutes on ice followed by two washes in flow buffer. The cells were fixed with Fix/Perm buffer for 15 minutes, washed with Perm buffer, then stained against Ki-67 (eBioscience) according to manufacturer's suggested protocol. Cells were either analyzed immediately or the following day
Instrument	Guava easyCyte 8HT (Millipore)
Software	guavaSoft 2.7
Cell population abundance	Glioma cells were typically 85-90% of the live cells to be analyzed (shown in Figure 2B). Approximate purity was ensured by labeling the glial cells with dyes and performing negative selection of the glioma cells prior to further analyses
Gating strategy	Cells were first identified on a plot of FSC vs SSC. This gate was applied to a plot of SSC-A vs SSC-H, and singlets were identified along the diagonal. The singlet-cells gate was applied to the plot of live/dead dye vs FSC or the corresponding histogram, and the non-stained cells were selected as 'live'. This gate of live-singlet-cells was applied to a plot of CellTracker vs Vybrant DiD, and the unstained glioma cells were selected. The final gate of glioma/live/singlet/cells was applied to all further plots or histograms for analysis. Positive antibody staining was identified using isotype contols.

- [x] Tick this box to confirm that a figure exemplifying the gating strategy is provided in the Supplementary Information.
